# Meta-analyses of visceral versus non-visceral metastatic hormone receptor-positive breast cancer treated by endocrine monotherapies

**DOI:** 10.1038/s41523-021-00222-y

**Published:** 2021-02-12

**Authors:** John F. R. Robertson, Angelo Di Leo, Stephen Johnston, Stephen Chia, Judith M. Bliss, Robert J. Paridaens, Jasmine Lichfield, Ian Bradbury, Christine Campbell

**Affiliations:** 1 Graduate Entry Medicine, University of Nottingham, School of Medicine, Nottingham, UK; 2 Sandro Pitigliani Medical Oncology Unit, Department of Oncology, Hospital of Prato, Instituto Toscano Tumori, Prato, Italy; 3 Royal Marsden NHS Foundation Trust, London, UK; 4 Department of Medical Oncology, British Columbia Cancer Agency, Vancouver, BC Canada; 5 Institute of Cancer Research, London, UK; 6 Department of Oncology, Universitair Ziekenhuis Gasthuisberg, Leuven, Belgium; 7 Global Medicines Development, AstraZeneca, Cambridge, UK; 8 Biostatistics, Frontier Science, Kincraig, Scotland UK; 9 Present Address: Eisai, Hatfield, UK

**Keywords:** Metastasis, Cancer therapy

## Abstract

Endocrine therapy (ET) is recommended as first-line therapy for the majority of patients with hormone receptor-positive (HR+), human epidermal growth factor 2-negative advanced breast cancer (ABC); however, the efficacy of ET in patients with visceral metastases (VM) versus patients whose disease is limited to non-visceral metastases (non-VM) is debated. Meta-analyses including available data from randomised controlled trials of first- and second-line endocrine monotherapies for patients with HR+ ABC were performed to address this question. In one and two-stage meta-analyses, progression-free survival (PFS), overall survival (OS), clinical benefit rate (CBR) and duration of clinical benefit (DoCB) outcomes were analysed. In the first-line meta-analysis (seven trials; *n* = 1988) tamoxifen and fulvestrant significantly improved PFS, OS and CBR for patients with non-VM versus those whose disease included VM. The most substantial hazard ratios were observed for fulvestrant 500 mg; 0.56 (95% confidence interval [CI] 0.45−0.70) and 0.55 (95% CI 0.42−0.72) for PFS and OS, respectively. In the second-line meta-analysis (seven trials; *n* = 2324), all ET combined was more effective (in terms of PFS, OS and DoCB) for non-VM versus VM. In both meta-analyses, patients with non-liver VM had better clinical outcomes than patients with liver VM for all types of ET. Patients whose disease included non-VM sites had better clinical outcomes with endocrine monotherapy compared with patients whose disease included VM. These findings may facilitate better informed treatment decision-making.

## Introduction

Breast cancer (BC) is the leading cause of cancer-related death for women globally^1^. The majority of BCs are hormone receptor-positive (HR+)^2^, and of those who develop metastatic disease, many develop visceral metastases (VM)^3^. In patients with HR+ advanced BC (ABC), those with VM are considered to have poorer prognosis than patients whose disease is limited to non-visceral metastases (non-VM)^4^.

In the absence of visceral crisis or concern over endocrine resistance, current guidelines recommend endocrine therapy (ET), including treatment with aromatase inhibitors (AIs; anastrozole, letrozole and exemestane), the selective estrogen receptor degrader (SERD) fulvestrant, or the selective estrogen receptor modulator (SERM) tamoxifen, either as monotherapy or in combination with cyclin-dependent kinase (CDK)4/6 inhibitors, as first-line therapy for postmenopausal patients with HR+, human epidermal growth factor 2-negative (HER2−) ABC^5,6^. However, the efficacy of ET in patients with VM compared with non-VM is debated^4^. Indeed most studies of ET include VM versus non-VM as a stratification factor and/or a subgroup analysis.

This meta-analysis aimed to ascertain where maximal benefit can be derived from endocrine monotherapy, by evaluating the clinical efficacy of different endocrine monotherapies in different patient subgroups; fulvestrant (a SERD), tamoxifen (a SERM) and AIs in the first- and second-line treatment of patients with HR+ ABC with VM and with non-VM. We wanted to test the following:

(I) Whether, in both first- and second-line settings, ET was more efficacious in patients whose disease involved non-VM sites, compared with those where VM sites were involved.
(II) If a difference was observed in (i), whether the response between patients with visceral non-liver metastases (VnLM) and patients with visceral liver metastases (VLM) was different.
(III) If a difference was observed in (ii), how does ET compare in non-VM and VnLM versus VLM?
(IV) We then wanted to assess whether any differences in (i) to (iii) above were generic to all ETs or specific to a particular class(es) of ET (i.e. SERD, SERM or AI).

## Results

### Study characteristics

**Fig. 1:**
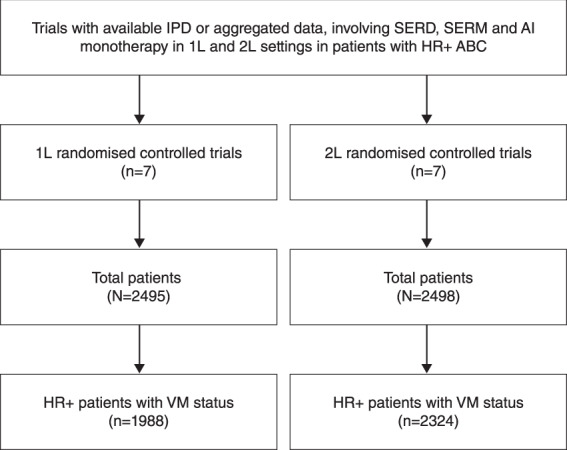
Study-selection flow chart. 1 L first line, 2 L second line, AI aromatase inhibitor, BC breast cancer, HR+ hormone receptor-positive, IPD individual patient data, n number of patients, SERD selective estrogen receptor degrader, SERM selective estrogen receptor modulator, VM visceral metastases.

In the first-line meta-analysis, 1988 patients had HR+ ABC with available VM status: 969 patients had HR+ BC involving VM; 1019 patients had HR+ ABC with non-VM (Fig. 1, Table 1). Of HR+ patients with known VM status, 691 (34.8%) were treated with SERM (tamoxifen), 805 (40.5%) with AI (8.4% exemestane, 32.1% anastrazole) and 492 (24.7%) with SERD (fulvestrant 500 mg) (Table 1) in the first-line setting. In the second-line meta-analysis, 2324 patients had HR+ ABC with VM status: 1271 patients had HR+ BC involving VM; 1053 patients had HR+ ABC with non-VM (Table 1). Of HR+ patients with known VM status treated in the second-line setting, 936 (40.3%) received AI (25.1% exemestane, 15.1% anastrazole), 1388 (59.7%) received SERD (49.7% fulvestrant 250 mg, 10.2% fulvestrant 500 mg) and none received SERM (Table 1). In the first-line setting, in trials with patient recruitment ending prior to January 2006, 59.4% of patients received SERM, 40.6% received AI and none received SERD, whereas in trials where patient recruitment ended after January 2006, 59.6% received SERD, 40.4% received AI and none received SERM (Table 1).

**Table 1:** Studies included and study arm characteristics.

Study	Treatment arms	Total patients n per arm	Patient age, median (range)	Patient enrolment dates	HR + patients with VM status	HR + non-VM n (% per arm)	HR + VM n (% per arm)	HR + VLM n (% per arm)	HR + VnLM n, (% per arm)
First line									
EORTC^35^	Tamoxifen	189	62 (37–87)	Oct 1996–Dec 2002	178	95 (53.4)	83 (46.6)	NA	NA
	Exemestane	182	63 (37–86)		168	88 (52.4)	80 (47.6)	NA	NA
Study 0025^36^	Tamoxifen	274	66 (43–92)	Nov 1998–Jun 2000	209	135 (64.6)	74 (35.4)	23 (11.0)	51 (24.4)
Study 0027^a ^^37^	Tamoxifen	328	66 (41–92)	Aug 1995–Jul 1998	144	71 (49.3)	73 (50.7)	15 (10.4)	58 (40.3)
	Anastrozole	340	67 (34–91)		153	98 (64.1)	55 (35.9)	24 (15.7)	31 (20.3)
Study 0030^38^	Tamoxifen	182	67 (40–92)	Feb 1996–Jul 1998	160	80 (50.0)	80 (50.0)	26 (16.3))	54 (33.8)
	Anastrozole	171	68 (30–88)		150	73 (48.7)	77 (51.3)	13 (8.7)	64 (42.7)
FIRST^8^	Anastrozole	103	68 (48–87)	Feb 2006–Jan 2008	102	45 (44.1)	57 (55.9)	13 (12.7)	44 (43.1)
	Fulvestrant 500 mg	102	66 (40–89)		100	54 (54.0)	46 (46.0)	14 (14.0)	32 (32.0)
FALCON^9^	Anastrozole	232	62 (36–90)	Oct 2012–Jul 2014	232	113 (48.7)	119 (51.3)	NA	NA
	Fulvestrant 500 mg	230	64 (38–87)		230	95 (41.3)	135 (58.7)	NA	NA
CONFIRM^b ^^39–41^	Fulvestrant 500 mg	162	61 (not reported)	Feb 2005–Aug 2007	162	72 (44.4)	90 (55.6)	47 (29.0)	43 (26.5)
Total^c^		2495			1988	1019 (51.3)	969 (48.7)		
	SERM				691 (34·8)				
	AI				805 (40·5)				
	SERD 500 mg				492 (24·7)				
Second line									
Study 0020^a ^^44^	Anastrozole	230	64 (33–89)	May 1997–Sep 1999	183	109 (59.6)	74 (40.4)	42 (23.0)	32 (17.5)
	Fulvestrant 250 mg	219	63 (35–86)		160	93 (58.1)	67 (41.9)	39 (24.4)	28 (17.5)
Study 0021^a ^^45^	Anastrozole	193	62 (36–94)	May 1997–Sep 1999	168	93 (55.4)	75 (44.6)	38 (22.6)	37 (22.0)
	Fulvestrant 250 mg	204	63 (33–89)		177	88 (49.7)	89 (50.3)	44 (24.9)	45 (25.4)
EFECT^46^	Exemestane	340	63 (32–91)	Aug 2003–Nov 2005	336	142 (42.3)	194 (57.7)	106 (31.5)	88 (26.2)
	Fulvestrant 250 mg	351	63 (38–88)		345	150 (43.5)	195 (56.5)	109 (31.6)	86 (24.9)
SoFEA^d ^^47^	Exemestane	249	66 (59–75)	Mar 2004–Aug 2010	249	104 (41.8)	145 (58.2)	72 (28.9)	73 (29.3)
	Fulvestrant 250 mg	231	63 (57–74)		231	88 (38.1)	143 (61.9)	72 (31.2)	71 (30.7)
FINDER1^48^	Fulvestrant 250 mg	45	61 (50–77)	Mar 2006–Mar 2008	43	19 (44.2)	24 (55.8)	9 (20.9)	15 (34.9)
	Fulvestrant 500 mg	47	61 (45–83)		45	19 (42.2)	26 (57.8)	9 (20.0)	17 (37.8)
FINDER2^49^	Fulvestrant 250 mg	47	63 (42–88)	May 2006–Jun 2008	46	13 (28.3)	33 (71.7)	19 (41.3)	14 (30.4)
	Fulvestrant 500 mg	46	67 (49–85)		45	9 (20.0)	36 (80.0)	19 (42.2)	17 (37.8)
CONFIRM^39–41^	Fulvestrant 250 mg	152	61 (not reported)	Feb 2005–Aug 2007	152	71 (46.7)	81 (53.3)	41 (27.0)	40 (26.3)
	Fulvestrant 500 mg	144	61 (not reported)		144	55 (38.2)	89 (61.8)	36 (25.0)	53 (36.8)
Total^c^		2498			2324	1053 (45.3)	1271 (54.7)	655 (28.2)	616 (26.5)
	AI				936 (40·3)				
	SERD 250 mg				1154 (59·7)				
	SERD 500 mg				234 (10·1)				

Superscript numerals indicate cited study references and superscript letters indicate footnotes.

*AI* aromatase inhibitor, *BC* breast cancer, *EORTC* European Organisation for Research and Treatment of Cancer, *HR+* hormone receptor-positive, *non-VM* non-visceral metastases, *SERD* selective estrogen receptor degrader, *SERM* selective estrogen receptor modulator, *VM* visceral metastases.

^a^Patient age data are mean and range.

^b^Patients from CONFIRM were treated in both the first- and second-line settings.

^c^Total number of patients with HR+ BC and VM status equals the number of HR+ patients with non-VM plus the number of patients with VM. Percentage of HR+ patients with non-VM, VM, VLM, VnLM equals number of HR+ patients with each status divided by total HR+ patients with known VM status per trial arm.

^d^Patient age data are median and interquartile range.

### Meta-analyses: ET for VM versus non-VM

**Fig. 2:**
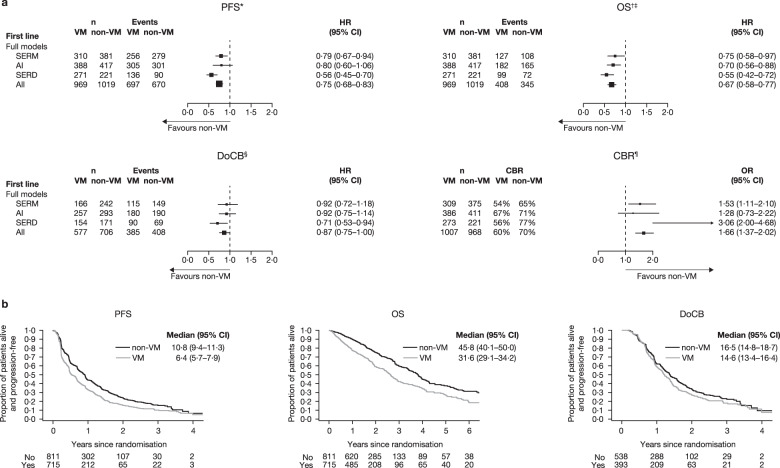
Clinical outcome measures for VM versus non-VM in the first-line setting. **a** Forest plots of PFS, OS, DoCB and CBR. ^*^Random effect for trial were fitted to AI and all data. ^†^Fixed effects for trial were fitted in all models. ^‡^OS data for Study 0027 are based on aggregated mature survival data. ^§^Fixed effect for trial was fitted in all models. ^¶^Fixed-effect model was fitted to the SERD, SERM and all data; random effects for trial were included in the models for AI. **b** Projected probability of PFS, OS and DoCB. Kaplan–Meier curves are for ET combined and do not include FALCON data. Median (95% CI) PFS/OS/DoCB in months. AI aromatase inhibitor, CBR clinical benefit rate, DoCB duration of clinical benefit, HR hazard ratio, non-VM non-visceral metastases, n number of patients, OS overall survival, PFS progression-free survival, SERD selective estrogen receptor degrader, SERM selective estrogen receptor modulator, VM visceral metastases. Statistics for full models: PFS: SERM: *p* = 0·008, heterogeneity test *p* = 0·26; AI: *p* = 0·122, heterogeneity test *p* < 0·05; SERD: *p* < 0·001, heterogeneity test *p* = 0·40; All: *p* < 0·001, heterogeneity test *p* < 0·05. OS: SERM: *p* = 0·031, heterogeneity test *p* = 0·21; AI: *p* = 0·002, heterogeneity test *p* = 0·24; SERD: *p* < 0·001, heterogeneity test *p* = 0·68; All: *p* < 0·001, heterogeneity test *p* = 0·24. DoCB: SERM: *p* = 0·527, heterogeneity test *p* = 0·57; AI: *p* = 0·446, heterogeneity test *p* = 0·33; SERD: *p* = 0·018, heterogeneity test *p* = 0·13; All: *p* = 0·04, heterogeneity test *p* = 0·28.CBR: SERM: *p* = 0·009, heterogeneity test *p* = 0·19, AI: *p* = 0·388, heterogeneity test *p* = 0·02; SERD: *p* < 0·001, heterogeneity test *p* = 0·78; All: *p* < 0·001, heterogeneity test *p* = 0·18.

In the first-line setting, all ETs combined demonstrated significantly longer PFS and OS in patients with non-VM versus VM (Fig. 2, see Supplementary Fig. 1a for individual data). CBR was also significantly higher for all ET combined in patients with non-VM compared with VM (Fig. 2, Supplementary Fig. 1a).

PFS was significantly longer in patients with non-VM versus those with VM with SERD 500 mg and SERM. For AIs, the HR showed a trend towards longer PFS for patients with non-VM versus those with VM, but the 95% CI crossed 1. Similarly, compared with patients with VM, CBR was significantly higher in patients with non-VM who received SERD 500 mg or SERM (Fig. 2). Despite a trend towards higher CBR, AIs did not significantly improve CBR in non-VM versus VM patients (Fig. 2), a finding that is in keeping with previous analysis^7^. All three types of treatment demonstrated significantly longer OS in patients with non-VM versus VM. For all ETs combined, DoCB in the first-line setting was significantly longer for non-VM versus VM (*p* = 0.044, upper CI = 0.996) (Fig. 2). SERD produced significantly longer DoCB in patients with non-VM whereas SERM and AI did not (Fig. 2, Supplementary Fig. 1a). The median PFS and OS with different ETs in the first-line setting are shown in Table 2.

**Table 2:** Median PFS and OS for first- and second-line ET in patients with HR + ABC^a^.

	VM				non-VM			
	AI	SERM	SERD	All ET	AI	SERM	SERD	All ET
*First line*^b^								
Median PFS, months (95% CI)								
EORTC + 0027 + 0030	8·9 (6·1–11·2)	5·6 (4·8–6·7)	NA	—	10·2 (8·5–12·9)	8·6 (6·0–10·7)	NA	—
FALCON + FIRST	13·2 (10·1–16·5)	NA	13·8 (10·8–16·5)	—	15·2 (11·5–19·3)	NA	25·9 (21·9–34·0)	—
Median OS, months (95% CI)								
EORTC + 0030	29·8 (23·5–45·5)	33·6 (22·5–58·7)	NA	—	38·5 (34·5–47·1)	45·5 (32·8–51·6)	NA	—
FALCON + FIRST	38·5 (32·2–49·1)	NA	35·7 (29·7–49·9)	—	51·7 (40·9–75·8)	NA	68·6 (50·0–81·8)	—

	VM				non-VM			
	AI	SERD 250 mg	SERD 500 mg	All ET	AI	SERD 250 mg	SERD 500 mg	All ET
*Second line*								
Median PFS, months (95% CI)	2·9 (2·8–3·2)	3·5 (3·0–3·9)	5·5 (4·1–8·2)	3·3 (3·0–3·6)	5·4 (4·0–5·8)	5·6 (5·2–6·1)	10·3 (6·1–13·8)	5·6 (5·4–5·9)
Median OS, months (95% CI)	22·8 (20·1–23·9)	20·9 (19·2–22·8)	28·8 (22·5–42·2)	22·3 (20·7–23·5)	24·3 (22·1–25·7)	26·1 (24·0–28·1)	35·4 (23·6–47·2)	25·6 (24·3–27·7)

*ABC* advanced breast cancer, *AI* aromatase inhibitor, *EORTC* European Organisation for Research and Treatment of Cancer, *ET* endocrine therapy, *HR+* hormone receptor-positive, *IPD* individual patient data, *NA* not applicable, *non-VM* non-visceral metastases, *OS* overall survival, *PFS* progression-free survival, *SERD* selective estrogen receptor degrader, *SERM* selective estrogen receptor modulator, *VM* visceral metastases.

^a^No formal statistical analysis was performed.

^b^It was not possible to generate the overall median PFS and OS for the first-line meta-analysis, medians are presented where IPD was available.

**Fig. 3:**
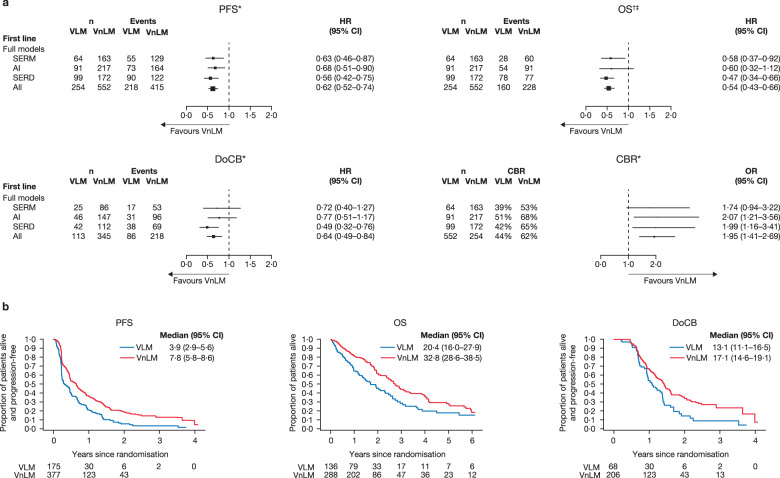
Clinical outcome measures for VLM versus VnLM in the first-line setting. **a** Forest plots of PFS, OS, DoCB and CBR. Data for VLM are not available for the EORTC trial. *Fixed effect for trial was fitted in all models. †Random effect for trial were fitted in model for AI. ‡OS data for Study 0027 are based on aggregated mature survival data. **b** Projected probability of PFS, OS and DoCB. Median (95% CI) PFS/OS/DoCB in months. AI aromatase inhibitor, CBR clinical benefit rate, DoCB duration of clinical benefit, HR hazard ratio, n number of patients, non-VM non-visceral metastases, PFS progression-free survival, OR odds ratio, OS overall survival, SERD selective estrogen receptor degrader, SERM selective estrogen receptor modulator, VLM visceral liver metastases, VM visceral metastases, VnLM visceral non-liver metastases. Statistics for full models: VLM vs VnLM PFS: SERM: *p* = 0·005, heterogeneity test *p* = 0·91; AI: *p* = 0·008, heterogeneity test *p* = 0·81; SERD: *p* < 0.001, heterogeneity test *p* = 0·95; All: *p* < 0·001, heterogeneity test *p* = 0·99. OS: SERM: *p* = 0·020, heterogeneity test *p* = 0·89; AI: *p* = 0·106, heterogeneity test *p* < 0·05; SERD: *p* < 0.001, heterogeneity test *p* = 0·34; All: *p* < 0·001, heterogeneity test *p* = 0·24. DoCB: SERM: *p* = 0·255, heterogeneity test *p* = 0·93; AI: *p* = 0·229, heterogeneity test *p* = 1·00; SERD: *p* < 0·001, heterogeneity test *p* = 0·08; All: *p* = 0·001, heterogeneity test *p* = 0·58. CBR: SERM: *p* = 0·077, heterogeneity test *p* = 0·72; AI: *p* = 0·008, heterogeneity test *p* = 0·43; SERD: *p* = 0·012, heterogeneity test *p* = 0·43; All: *p* < 0·001, heterogeneity test *p* = 0·80.

In patients with VnLM versus VLM, all ETs combined demonstrated significantly greater PFS, OS, DoCB and CBR in patients with VnLM (Fig. 3, see Supplementary Fig. 2a for individual data). Individually, SERD and SERM, were significantly better in terms of PFS and OS in patients with VnLM, compared with patients with VLM. SERD also produced significantly longer DoCB in patients with VnLM. AIs had a hazard ratio in favour of patients with VnLM that was similar to SERD and SERM, although the confidence intervals were wider and crossed one. CBR was broadly comparable between ETs (Fig. 3).

**Fig. 4:**
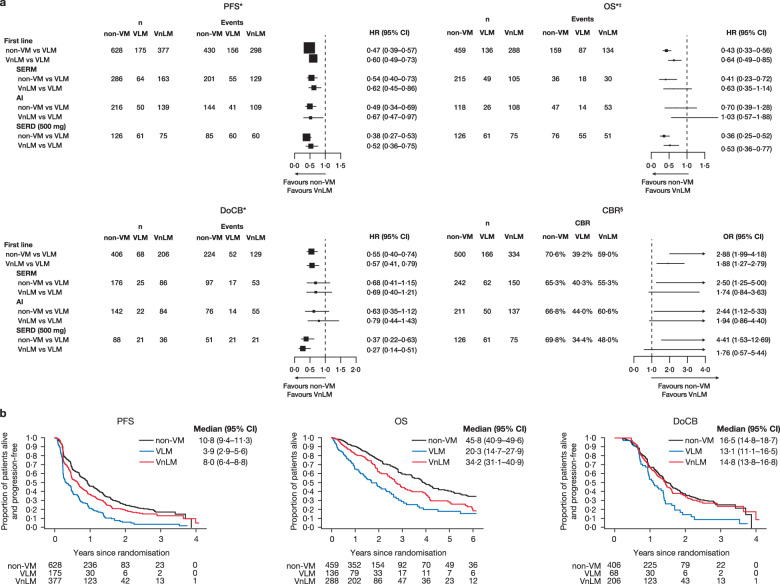
Clinical outcome measures for non-VM and VnLM versus VLM in the first-line setting. **a** Forest plots of PFS, OS, DoCB and CBR. Data for VLM are not available for the EORTC trial. *Fixed effect for trial was fitted in all models. ‡OS data for Study 0027 are based on aggregated mature survival data. §“one−stage” fixed−effects logistic regression models. **b** Projected probability of PFS, OS and DoCB. Median (95% CI) PFS/OS/DoCB in months. AI aromatase inhibitor, CBR clinical benefit rate, DoCB duration of clinical benefit, HR hazard ratio, n number of patients, non-VM non-visceral metastases, PFS progression-free survival, OR odds ratio, OS overall survival, SERD selective estrogen receptor degrader, SERM selective estrogen receptor modulato, VLM visceral liver metastases, Non-VM non-visceral metastases, VnLM visceral non-liver metastases. Statistics for full models: VLM vs VnLM vs non-VM. PFS: SERM: Interaction test *p* = 0.55; AI: Interaction test *p* = 0.25; SERD: Interaction test *p* = 0.22; All: Interaction test *p* = 0.29. OS: SERM: Interaction test *p* = 0.42; AI: Interaction test *p* = 0.06; SERD: Interaction test *p* = 0.54; All: Interaction test *p* = 0.25. DoCB: SERM: Interaction test *p* = 0.81; AI: Interaction test *p* = 0.54: SERD: Interaction test *p* = 0.07; All: Interaction test *p* = 0.07. CBR: SERM: Interaction test *p* = 0.26; AI: Interaction test *p* = 0.05; SERD: Interaction test *p* = 0.85; All: Interaction test *p* = 0.46.

Considering the third objective (VLM vs VnLM and non-VM), the HRs for PFS and OS in the overall analysis were more substantial in patients with non-VM and patients with VnLM, compared to VLM (Fig. 4); this trend was reproduced in virtually every individual study (Supplementary Fig. 3a). The Kaplan–Meier curves for PFS and OS across patients in the first-line setting suggest a hierarchy of disease prognosis with non-VM having better outcomes than VnLM which is, in turn, better than VLM (Fig. 4b).

**Fig. 5:**
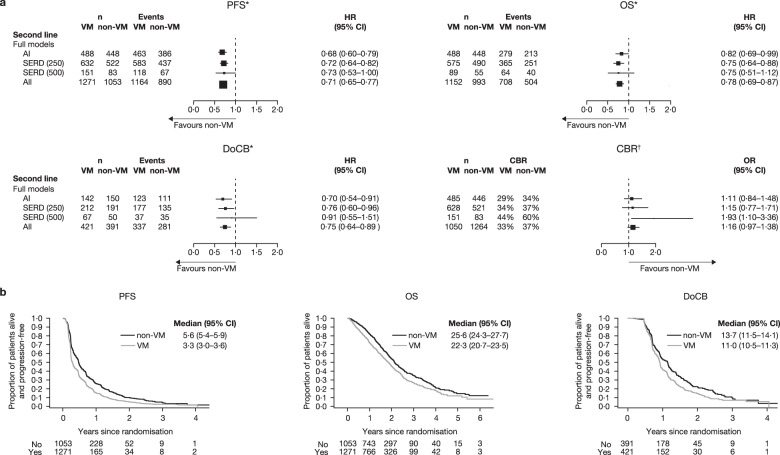
Clinical outcomes for VM versus non-VM in the second-line setting. **a** Forest plots of PFS, OS, DoCB and CBR. ^*^Fixed effect for trial was fitted in all models. ^†^Fixed-effect model was fitted to the SERD 250 mg, SERD 500 mg and all data; random effects for trial were included in the models for AI. **b** Projected probability of PFS, OS and DoCB. Median (95% CI) PFS/OS/DoCB in months. AI aromatase inhibitor, CBR clinical benefit rate, DoCB duration of clinical benefit, HR hazard ratio, n number of patients, non-VM non-visceral metastases, PFS progression-free survival, OR odds ratio, OS overall survival, SERD selective estrogen receptor degrader, SERM selective estrogen receptor modulator, VLM visceral liver metastases, VM visceral metastases, VnLM visceral non-liver metastases. Statistics for full models:PFS: AI: *p* < 0·001, heterogeneity test *p* = 0·13; SERD (250 mg): *p* < 0·001, heterogeneity test *p* = 0·08; SERD (500 mg): *p* = 0·048, heterogeneity test *p* = 0·83; All: *p* < 0·001, heterogeneity test *p* = 0·17. OS: AI: *p* = 0·036, heterogeneity test *p* = 0·35; SERD (250 mg): *p* < 0·001, heterogeneity test *p* = 0·95; SERD (500 mg): *p* = 0·161, heterogeneity test *p* = 1·00; All: *p* < 0·001, heterogeneity test *p* = 0·86. DoCB: AI: *p* = 0·007, heterogeneity test *p* = 0·40; SERD (250 mg): *p* = 0·023, heterogeneity test *p* = 0·08; SERD (500 mg): *p* = 0·720, heterogeneity test *p* = 0·31; All: *p* < 0·001, heterogeneity test *p* = 0·19. CBR: AI: *p* = 0·454, heterogeneity test *p* = 0·87; SERD (250 mg): *p* = 0·503, heterogeneity test *p* = 0·03; SERD (500 mg): *p* = 0·021, heterogeneity test *p* = 0·52; All: *p* = 0·109, heterogeneity test *p* = 0·10.

In the second-line setting, all ET combined was more effective in patients with non-VM compared with patients with VM, in terms of PFS, OS and DoCB (Fig. 5, see Supplementary Fig. 1b for individual data). AI and SERD 250 mg reached statistical significance for all three endpoints; for SERD 500 mg, the HRs were similar to AI and SERD 250 mg for OS and PFS but—with the smaller number of patients—the 95% CI ranges were larger and the upper limit exceeded 1 for OS and DoCB.

**Fig. 6:**
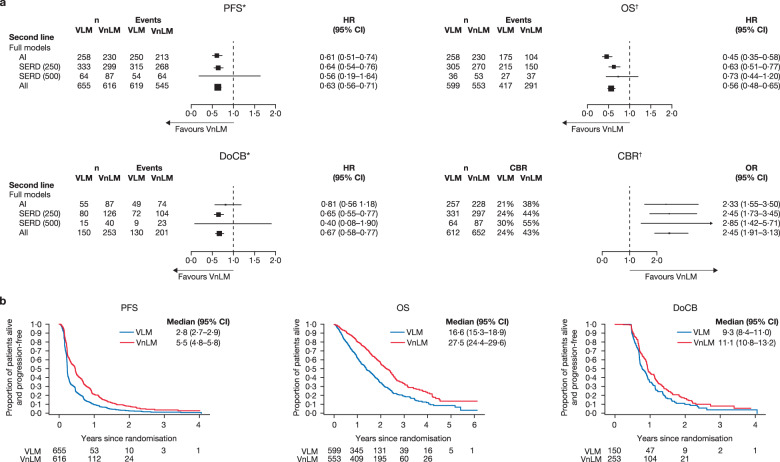
Clinical outcomes for VLM versus VnLM in the second-line setting. **a** Forest plots of PFS, OS, DoCB and CBR. ^*^Random effects for trial were fitted in SERD (500) model. ^†^Fixed effect for trial was fitted in all models. **b** Projected probability of PFS, OS and DoCB. Median (95% CI) PFS/OS/DoCB in months. AI aromatase inhibitor, CBR clinical benefit rate, DoCB duration of clinical benefit, HR hazard ratio, n number of patients, non-VM non-visceral metastases, PFS progression-free survival, OR odds ratio, OS overall survival, SERD selective estrogen receptor degrader, SERM selective estrogen receptor modulator, VLM visceral liver metastases, VM visceral metastases, VnLM visceral non-liver metastases. Statistics for full models: PFS: AI: *p* < 0·001, heterogeneity test *p* = 0·12; SERD (250 mg): *p* < 0·001, heterogeneity test *p* = 0·93; SERD (500 mg): *p* = 0·286, heterogeneity test *p* < 0·05; All: *p* < 0·001, heterogeneity test *p* = 0·20. OS: AI: *p* < 0·001, heterogeneity test *p* = 0·78; SERD (250 mg): *p* < 0·001, heterogeneity test *p* = 0·95; SERD (500 mg): *p* = 0·212, heterogeneity test *p* = 1·00; All: *p* < 0·001, heterogeneity test *p* = 0·66. DoCB: AI: *p* = 0·283, heterogeneity test *p* = 0·66; SERD (250 mg): *p* < 0·001, heterogeneity test *p* = 0·90; SERD (500 mg): *p* = 0·247, heterogeneity test *p* < 0·05; All: *p* < 0·001, heterogeneity test *p* = 0·35. CBR: AI: *p* < 0·001, heterogeneity test *p* = 0·72; SERD (250 mg): *p* < 0·001, heterogeneity test *p* = 0·98; SERD (500 mg): *p* = 0·003, heterogeneity test *p* = 0·17; All: *p* < 0·001, heterogeneity test *p* = 0·93.

Patients treated with second-line ET had better outcomes, in terms of PFS, OS, DoCB and CBR, if they had VnLM than if they had VLM (Fig. 6, Supplementary Fig. 2b). Compared with patients with VLM, patients with VnLM had a PFS and OS advantage with AI and SERD 250 mg, a DoCB advantage with SERD 250 mg and a CBR advantage with AI, SERD 250 mg and SERD 500 mg (Fig. 6).

**Fig. 7:**
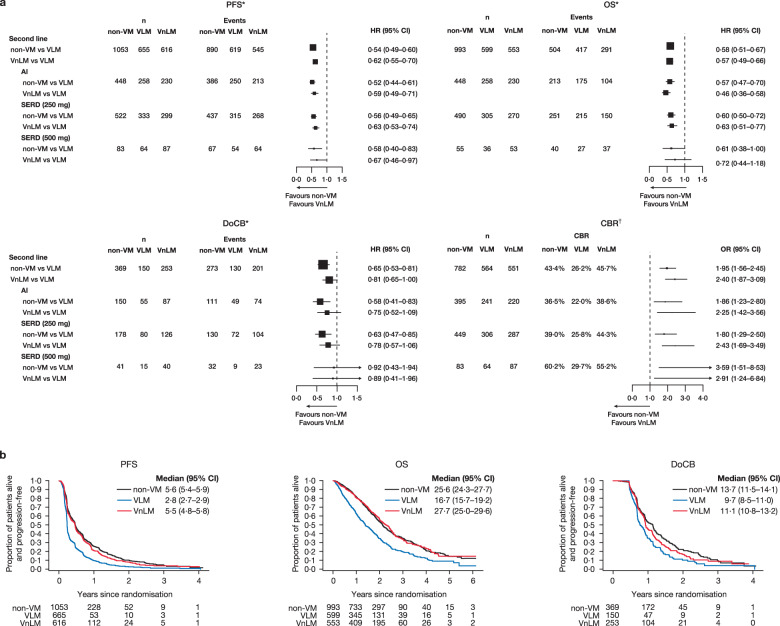
Clinical outcomes for non-VM and VnLM versus VLM in the second-line setting. **a** Forest plots of PFS, OS, DoCB and CBR. ^*^Fixed effect for trial was fitted in all models. ^†^Fixed-effect model was fitted to the AI SERD 250 mg and SERD 500 mg data; random effects for trial were included in the models for all data. §“one−stage” fixed−effects logistic regression models. **b** Projected probability of PFS, OS and DoCB. Median (95% CI) PFS/OS/DoCB in months. AI aromatase inhibitor, CBR clinical benefit rate, DoCB duration of clinical benefit, HR hazard ratio, n number of patients, non-VM non-visceral metastases, PFS progression-free survival, OR odds ratio, OS overall survival, SERD selective estrogen receptor degrader, SERM selective estrogen receptor modulator, VLM visceral liver metastases, Non-VM non-visceral metastases, VnLM visceral non-liver metastases. VLM vs VnLM vs non-VM. PFS: AI: Interaction test *p* = 0.15; SERD (250): Interaction test *p* = 0.18; SERD (500): Interaction test *p* = 0.05; All: Interaction test *p* = 0.06. OS: AI: Interaction test *p* = 0.66; SERD (250): Interaction test *p* = 0.98; SERD (500): Interaction test *p* = 0.98; All: Interaction test *p* = 0.78. DoCB: AI: Interaction test *p* = 0.59; SERD (250): Interaction test *p* = 0.10; SERD (500): Interaction test *p* = 0.11; All: Interaction test *p* = 0.03.CBR: AI: Interaction test *p* = 0.90; SERD (250): Interaction test *p* = 0.20; SERD (500): Interaction test *p* = 0.33; All: Interaction test *p* = 0.47.

A comparison of VLM versus VnLM and non-VM in the second-line setting found a PFS and OS advantage for all treatments in patients with non-VM and VnLM versus patients with VLM (Fig. 7, Supplementary Fig. 3b); this was reproducibly seen in virtually every study. As with the first-line setting, both non-VM and VnLM appear to do better than VLM in the second-line setting but there is no suggestion of a hierarchy of benefit between non-VM and VnLM for PFS and OS in the second-line setting.

## Discussion

The efficacy of ET in patients with VM and non-VM has been debated^4^. This may be due to lack of clarity in previous publications; for instance, some may have reported on VM versus non-VM while others have reported on VLM versus VnLM. Overall, our two meta-analyses, which to our knowledge are the largest reported on the topic, indicate that patients on endocrine monotherapy with non-VM have better clinical outcomes compared with patients with VM. These meta-analyses are also the largest to have divided VM into VLM and VnLM and show that these subgroups differ in their responsiveness to ET. This is clinically relevant in terms of selecting therapies, especially when considering endocrine monotherapy versus combination therapy.

In the first-line, both ER-blocking agents (tamoxifen [SERM] and fulvestrant [SERD]) demonstrated significantly better PFS, OS and CBR in patients with non-VM versus patients with VM. SERD also produced significantly longer DoCB in patients with non-VM. For AIs, the OS was statistically significantly better for non-VM versus VM patients and although the PFS did not reach statistical significance, the hazard ratio was similar to tamoxifen, suggesting that the effects of AIs are consistent with SERM. In the meta-analysis, all three endpoints (PFS, OS and CBR), had greater treatment effects for non-VM versus VM patients and the HRs showed greater benefit for fulvestrant 500 mg compared with SERM or AIs. Although our new meta-analyses do not, by themselves, allow us to conclude that SERD produces greater benefits in patients with non-VM than SERM or AIs, the results are in keeping with previously published data showing a statistically significant improvement in outcomes with fulvestrant 500 mg compared with anastrozole in both the FALCON and FIRST studies^7–11^. Indeed, the PFS analysis between fulvestrant and anastrazole for VM versus non-VM has been previously reported for FIRST and FALCON individually and in both, fulvestrant gave statistically significantly longer PFS compared to AI in the non-VM group but not the VM group^7^. These analyses imply that efficacy may be both disease site- and endocrine agent-dependent.

To account for these differences between non-VM and VM, and between the efficacy of ER-blocking agents compared to AIs, one possibility is that the distribution of luminal A and luminal B BC may be different between patients with non-VM versus VM and that the receptor-blocking agents are more effective on one of the two luminal subtypes. Alternatively, differential *ESR1* mutations could potentially have contributed to the differential response to ET according to visceral status, particularly in the second-line. However, this latter explanation is not relevant to the FALCON trial, where all patients were ET-naïve, or the FIRST trial, where approximately three-quarters of patients were ET-naïve and the majority of those who had received ET had received prior adjuvant tamoxifen^9,10^. An alternative explanation for the difference would be the distinct mechanisms of action of these agents; AIs reduce estradiol, the ligand for the ER, while ER-blocking agents bind to the ER. Furthermore, fulvestrant not only competes for estradiol-binding to the ER, but also degrades the receptor^12–14^.

This meta-analysis demonstrates that for patients with non-VM, modern optimized ET, even without the addition of CDK4/6 inhibitors, can achieve good outcomes. For example, in the combined FALCON and FIRST trials, the median PFS with fulvestrant 500 mg was 25.9 months and OS was 68.6 months (Table 2). The PFS and OS results in patients with non-VM and fulvestrant 500 mg in this meta-analysis may identify a subgroup of patients with a long survival on fulvestrant 500 mg monotherapy.

Patients with VnLM had significantly better PFS and OS with tamoxifen and fulvestrant 500 mg compared with patients with VLM: a similar significant benefit in PFS was seen with AIs and a similar, but non-significant, HR for OS. Patients with HR+ VnLM appear to respond well to ETs, again particularly when treated with fulvestrant 500 mg monotherapy. This is similar to results reported in a recent single centre study that analysed 398 patients with HR+, HER2− metastatic BC by site of disease who had been treated by fulvestrant 500 mg: the median PFS was similar in patients with non-VM and those with lung (without liver) metastases, while patients with liver metastases had significantly worse PFS^15^. One possible explanation we have looked at, but found no evidence for, was the possibility that a higher percentage of patients with visceral liver metastases were HER2+. In a large RCT, stratified by HER2 status, HR+/HER2+ tumours responded less well to ET alone than HER2- tumours^16^. In the same trial, and another RCT comparing an AI versus AI plus an anti-HER2 therapy, it has been reported that addition of an anti-HER2 targeting agent increases CBR and PFS compared ET alone in HER2+ tumours but not HER2- tumours^16–18^. Although HER2 status was not known in five of seven trials in the first-line and four of seven trials in the seocnd line setting, we have no evidence to suggest that differences in the number of HER2+ patients between trials had significant effects on overall findings of VLM versus VnLM. In fact, in the studies included in our meta-analysis where HER2 status was known, less than 10% of patients with ER+ tumours were also HER2+, a finding consistent with numerous previous studies^19,20^. This overall figure of 10% includes the 18% of patients in the FIRST trial who were reported to be HER2+/3+ by immunohistochemistry (IHC). Since the majority of HER2 (2+, IHC) are negative by FISH, this would mean that the percentage of patients that are truly HER2-positive was smaller than 10%.

The present meta-analysis shows that in the first-line, patients with HR+ ABC with non-VM and VnLM, who form the majority of the patient population, have significantly better outcomes on ET than patients with VLM. Notably, the latter VLM group represent only a small subgroup of the overall population of patients included in this analysis (*n* = 175/1732 patients where VLM vs VnLM status was known [10%] HR+ patients, Table 1). Therefore, the site of metastasis—particularly lack of liver involvement—may be one key factor to bear in mind when selecting the type of first-line ET, i.e. monotherapy or combination therapy, along with the other factors described by ESO-ESMO guidelines^5^.

The reasons for poorer outcomes in patients with liver metastases have not yet been established. Presence of liver metastases may indicate major changes in the tumour biology, with the implication that biopsy of liver lesions for immunophenotyping may be important for optimising treatment choice. An additional explanation of course is that liver involvement in some patients merely reflects the metastatic burden, which is difficult to quantify and could not be accounted for in this meta-analysis.

Patients in the VLM subgroup may still be prescribed ET in certain situations, providing frequent monitoring of response to detect early progression. Our present findings also indicate that, for postmenopausal patients with HR+, HER2− and VLM, combining ET with CDK4/6 inhibitors may be the best option, in view of the short PFS and OS to be expected with endocrine monotherapy. However, chemotherapy seems unavoidable in cases of VLM with life-threatening visceral crisis.

The results of this meta-analysis affirm the role of fulvestrant monotherapy in the first-line setting as a treatment option for HR+ ABC, particularly in patients with non-VM.

Fulvestrant may also be a favourable candidate for combination with CDK4/6 inhibitors for the first-line treatment of HR + ABC, especially given the significant OS result in the FIRST study^10^. Supporting this particular combination are the results of the MONALEESA-3 study^21,22^, where median OS and PFS were significantly improved with fulvestrant plus ribociclib, compared with placebo plus fulvestrant (results in the overall and first-line populations were consistent)(Supplementary Table 1).

In this meta-analysis of second-line ET for postmenopausal patients with HR+ ABC, all types of treatment combined were more effective for PFS, OS and DoCB in patients with non-VM compared with patients with VM. Although the HRs were similar for all types of ET, only AIs and SERD 250 mg reached statistical significance. This difference may be accounted for by the smaller patient numbers in the SERD 500 mg group, given the HRs were similar, but with wider CIs.

Overall, data in the second-line setting were more consistent between the three ETs than results in the first-line meta-analysis, with the caveat of low patient numbers for the SERD 500 mg group. Patients receiving ET in the second-line are generally less sensitive to ET, unless they were selected based on prior sensitivity. As such, it is often more difficult to detect significant differences between endocrine agents than in the first-line setting.

For both subgroups of patients, SERD 500 mg produced the longest median PFS and OS. Remaining mindful of the caveats of cross-trial comparisons, for patients with non-VM, median PFS and OS (10.3 and 35 months, Table 2) for those receiving SERD 500 mg monotherapy were similar to those reported for combination therapy (palbociclib plus fulvestrant) in the PALOMA-3 study^23,24^. Clinical outcomes in terms of PFS and OS for second-line patients with non-VM were generally improved compared with patients with VM. When VM were subdivided into VLM and VnLM, then both VnLM and non-VM did significantly better than VLM, with no difference seen between VnLM and non-VM overall for all ETs combined or for any individual type of ET (i.e. SERM, AI or SERD). In this meta-analysis VLM comprised 28% of patients (*n* = 655/2324) in the second-line setting.

Current guidelines suggest the combination of a CDK4/6 inhibitor with an AI or fulvestrant as a treatment option for patients with HR+, HER2− ABC with progression following prior ET^5,25^. Fulvestrant has been approved by the US Food and Drug Administration and the European Medicines Agency in combination with palbociclib, ribociclib or abemaciclib^26–31^. The results presented here suggest that patients with a better prognosis (non-VM and VnLM) may still be considered for endocrine monotherapy—in particular fulvestrant 500 mg—especially if they responded to prior ET, as opposed to combination therapy. Patients with poor prognosis who do not show significant benefit from endocrine monotherapy may be more appropriate for ET in combination with a CDK4/6 inhibitor. Notably, the PALOMA-3 study reported no significant increase in OS for patients with HR+, HER2− ABC who received palbociclib plus fulvestrant versus fulvestrant alone^23^, although the MONARCH 2 study recently reported a significant improvement in OS with fulvestrant plus abemaciclib versus fulvestrant alone (Supplementary Table 1)^32^.

Overall, these results reinforce what many clinicians already believed, that patients with non-VM have better outcomes than those with VM. More clearly than has been shown previously, this meta-analysis has highlighted that within the group of patients with VM those with VnLM do better than those with VLM. The VLM group seems to be the least sensitive to ET, with potential clinical implications. The Kaplan–Meier curves for PFS, OS and DoCB demonstrate very clearly the differences between patients with non-visceral metastases and those with liver or non-liver metastases for both first and second-line ET and help identify a group where endocrine monotherapy may be considered as a good initial treatment.

As far as the type of ET is concerned, our first-line meta-analysis suggested a superiority of fulvestrant 500 mg over the other ETs analysed, which is supported by direct comparisons in the FIRST/FALCON trials. This was less evident in the second-line, where there are fewer hormone-sensitive patients, making it more difficult to differentiate between ETs in this setting.

The results of the present meta-analyses may facilitate better informed treatment decision-making by taking into account the metastatic site and line of treatment.

## Methods

### Study selection

Anonymised, individual patient data (IPD) or aggregated data were obtained from studies involving SERD, SERM and AI monotherapy in the first- and second-line settings in patients with HR+ ABC: each trial had ethical approval and informed consent for use of the data. Studies with available IPD or aggregated data were included (Fig. 1). A literature search was performed to identify randomized trials of first-line mono-endocrine therapy in metastatic breast cancer from 1995 onwards. The results of the search included 5 phase 3 trials of a third-generation aromatase inhibitor (AI) versus tamoxifen. One of the five studies^33^ identified was excluded as an outlier based on a significantly lower PFS HR (0.13) and upper 95% CI limit (0.20) compared to the other four trials. Randomized trials of fulvestrant versus an AI were also searched. One study^34^, SWOG 0226, of fulvestrant 250 mg plus AI versus AI alone could not be included as the study group declined to share the data. There were two randomized trials of fulvestrant 500 mg versus and an AI—the FIRST and FALCON trials—both of which were included.

For the Phase 3 trials of mono-endocrine therapy in the second-line setting the literature search was limited to the trials involving fulvestrant versus an AI: seven trials were identified and all were included

In the first-line setting, seven randomised controlled trials (RCTs) were included in the meta-analysis (Fig. 1, Table 1), including five double-blind, placebo-controlled RCTs^8,9,35–41^. Letrozole Study PO25 data^42,43^ (letrozole *vs* tamoxifen) were requested but not included, as IPD and analyses by visceral disease status were unavailable.

Aggregated data for the FALCON study were used in the first-line meta-analyses as the study is ongoing; since IPD were not available, Kaplan–Meier plots for progression-free survival (PFS) and overall survival (OS) from the FALCON and FIRST studies combined (i.e. fulvestrant 500 mg [SERD] *vs* anastrozole [AI]) were provided by AstraZeneca. For Study 0027, aggregate mature OS data were used for comparisons of non-VM versus VM, and VLM versus VnLM. Mature OS data for Study 0027 were not included in the comparison of VLM versus VnLM and non-VM as we did not have consent to use some of the patients’ data.

In the second-line setting, studies included in the meta-analysis are shown in Table 1^44–49^. For OS, there were no deaths for the FINDER1 and FINDER2 studies. Approximately half of patients in the EFECT study (fulvestrant 250 mg vs exemestane) received the treatments as third-line ET, which would slightly reduce the PFS and OS^46^.

### Patients

Postmenopausal women with HR+ (estrogen receptor [ER] or progesterone receptor-positive), locally advanced or metastatic BC were included. The HR status of patients in these trials was determined locally, without central confirmation. Patients were treated in the first-line (defined as no prior ET for ABC; although adjuvant ET or ET for early BC was permitted in some trials) or second-line setting (relapse or progression on, or following, prior ET). Two of seven trials included in the first-line meta-analysis (FIRST, FALCON), and three of seven trials included in the second-line meta-analysis (SoFEA, FINDER1, FINDER2) reported tumours HER2 status. The mean percentage of HER2-positive tumours across these trials combined was 7.8 (range 0–18.6)%.

### Meta-analyses

As per the FALCON study, VM in these meta-analyses included baseline disease at any of the following sites: adrenal, bladder, central nervous system, oesophagus, liver, lung, peritoneum, pleura, renal, small bowel, stomach, pancreas, thyroid, colon, rectal, ovary, biliary tract, ascites, pericardial effusion, spleen or pleural effusion (including presence or absence of disease at other non-visceral sites)^9^. Non-VM was classified as disease not involving VM sites.

Two two-stage meta-analyses were used to analyse the clinical benefit rate (CBR), duration of clinical benefit (DoCB), PFS and OS between patients with VM and non-VM by type of therapy, within both the first- and second-line settings.

Recruitment to these RCTs spanned 19 years. AIs were randomised against tamoxifen in three studies between August 1995 and December 2002, and against fulvestrant 500 mg between February 2006 and July 2014. The median PFS and OS for AIs were different in these two time periods. Table 2 details the randomised comparisons between the AI and SERM, and AI and SERD classes for the two time periods, with AIs acting as a way of comparing all three types of ET. The most rational explanation for the increased median PFS and OS for the AIs would appear to be that the patient populations differed over two decades.

The Statistical Analysis Plan prospectively defined the comparisons to be: (i) non-VM versus VM; (ii) VLM versus VnLM; and if these looked different, then (iii) VLM versus VnLM and non-VM were to be compared. The Peto method for pooled odds ratios (ORs) was used to calculate *p*-values, ORs and confidence intervals (CIs) for CBR. The Yusef–Peto method was used to calculate *p*-values, hazard ratios (HRs), and CIs for PFS, OS and DoCB. Significance was tested at 5% (two sided).

Heterogeneity was assessed for the one-stage logistic regression and Cox models by testing the interaction between metastasis type and trial; random effects were fitted where there was evidence of heterogeneity. In the two-stage meta-analysis, a random effect model for trial was included when there was evidence of heterogeneity (using Tarone’s test or Cochran’s Q); otherwise, fixed-effect models were generated. One-stage IPD meta-analyses were used to analyse survival endpoints and test the three-level dummy variables (i.e. VLM vs VnLM and non-VM).

### Role of the funding source

The funder of the meta-analyses was the University of Nottingham, which was responsible for study design, data collection, data analysis, data interpretation and writing of the report. The corresponding author had full access to all the data in the study and had final responsibility for the decision to submit for publication.

### Reporting summary

Further information on research design is available in the Nature Research Reporting Summary linked to this article.

## Supplementary information

Supplementary Materials

Reporting Summary Checklist

## Data Availability

The file names and descriptions for all the original clinical trial data used in the meta-analyses, are available in the following metadata record: 10.6084/m9.figshare.13292621^50^. The datasets that support the findings of this study are not publicly available, but will be made available upon reasonable request from the corresponding author, Prof John Robertson, email address: John.Robertson@nottingham.ac.uk. The data may be obtained upon request for specific use as long as the request is in keeping with the terms of the agreement under which the University of Nottingham received the data. In accordance with the agreement under which Nottingham University gained approval to use the data, permission for sharing the data beyond those permitted in the agreement is not approved. The SAS analysis file, will not be made available, as a condition of the agreement to obtain the data was that they be encrypted during the work and removed completely from the encrypted workspace once the project was completed.

## Code availability

No custom code was used. Data analysis was performed using standard R (v3.5.3) functions from the ‘Metafor’ package and standard SAS (v9.4) survival functions, specifically, PROC PHREG and PROC GLIMMIX.
