# Supplementary material for: Meta-analyses of visceral versus non-visceral metastatic hormone receptor-positive breast cancer treated by endocrine monotherapies
Source: NPJ Breast Cancer. 2021 Feb 12;7:11. doi: 10.1038/s41523-021-00222-y (PMC7881093; doi:10.1038/s41523-021-00222-y)
Supplement: Supplementary file 1 — Supplementary Materials [file 41523_2021_222_MOESM1_ESM.pdf]

Supplementary Table 1. Median PFS and OS in previously published trials

			**Median PFS, months (95% CI)**	HR	**Median OS, months (95% CI)**	HR
**FALCON**[a 1]	All patients	Fulvestrant	16·6	0·797 (95% CI 0·637–0·999; p=0·0486)	-	0.88 (95% CI 0.63–1.22, p=0.43)
		Anastrozole	13·8		-	
	VM	Fulvestrant	13·8	0·99 (95% CI 0·74–1·33)	-	-
		Anastrozole	15·9		-	
	Non-VM	Fulvestrant	22·3	0·59 (95% CI 0·42–0·84)	-	-
		Anastrozole	13·8		-	
**FIRST** [2–4]	All patients	Fulvestrant	23·4	0·66 (95% CI 0·47–0·92; p=0·01)	54·1	0·70 (95% CI 0·50–0·98; p=0·04)
		Anastrozole	13·1		48·4	
	VM	Fulvestrant	9·8	0·82 (95% CI 0·54–1·26)	32·1	0·86 (95% CI 0·56–1·34)
		Anastrozole	9·9		38·5	
	Non-VM	Fulvestrant	34·0	0·58 (95% CI 0·34–0·99)	76·6	0·68 (95% CI 0·40–1·18)
		Anastrozole	21·3		60·9	
**PALOMA-1** [5]	All patients	Palbociclib plus letrozole	20·2	0·488 (95% CI 0·319–0·748; p=0.0004)	37.5	0.90 (95% CI 0.62–1.29; p=0.28)
		Letrozole monotherapy	10·2		34.5	
**PALOMA-2** [6]	All patients	Palbociclib plus letrozole	24·8	0·58 (95% CI 0·46–0·72; p<0·001)	-	-
		Letrozole monotherapy	14·5		-	-
	VM	Palbociclib plus letrozole	-	0·63 (95% CI 0·47–0·85)	-	-
		Letrozole monotherapy	-		-	-
	Non-VM	Palbociclib plus letrozole	-	0·50 (95% CI 0·36–0·70)	-	-
		Letrozole monotherapy	-		-	-
**PALOMA-3** [7,8]	All patients	Palbociclib plus fulvestrant	9·2	0·42 (95% CI 0·32–0·56; p<0·001)	34·9	0·81 (95% CI 0·64–1·03; p=0·09)
		Fulvestrant plus placebo	3·8		28·0	
	VM	Palbociclib plus fulvestrant	-	0·45 (95% CI 0·32–0·63)	27·6	0·85 (95% CI 0·64–1·13)

		Fulvestrant plus placebo	-		24·7	
	Non-VM	Palbociclib plus fulvestrant	-	0·36 (95% CI 0·22–0·60)	-	-
		Fulvestrant plus placebo	-		-	-
**MONARCH-2** [9]	All patients	Fulvestrant plus abemaciclib	16·4	0·553 (95% CI 0·449–0·681; p<0·001)	46·7	0·757 (95% CI 0·606–0·945; p=0·01)
	All patients	Fulvestrant plus placebo	9·3		37·3	
**MONALEESA-3** [10,11]	All patients	Fulvestrant plus ribociclib	20·5	0·593 (95% CI 0·480–0·732; p<0·001)	NR	0·724 (95% CI 0·568–0·924; p=0·00455)
		Fulvestrant plus placebo	12·8		40·0	
	First-line	Fulvestrant plus ribociclib	33·6	0·546 (95% CI 0·415–0·718)	NR	0·700 (95% CI 0·479–1·021)
		Fulvestrant plus placebo	19·2		45·1	

[a]Kaplan Meier curves and median overall survival could not be calculated due to insufficient follow-up time (31% maturity).

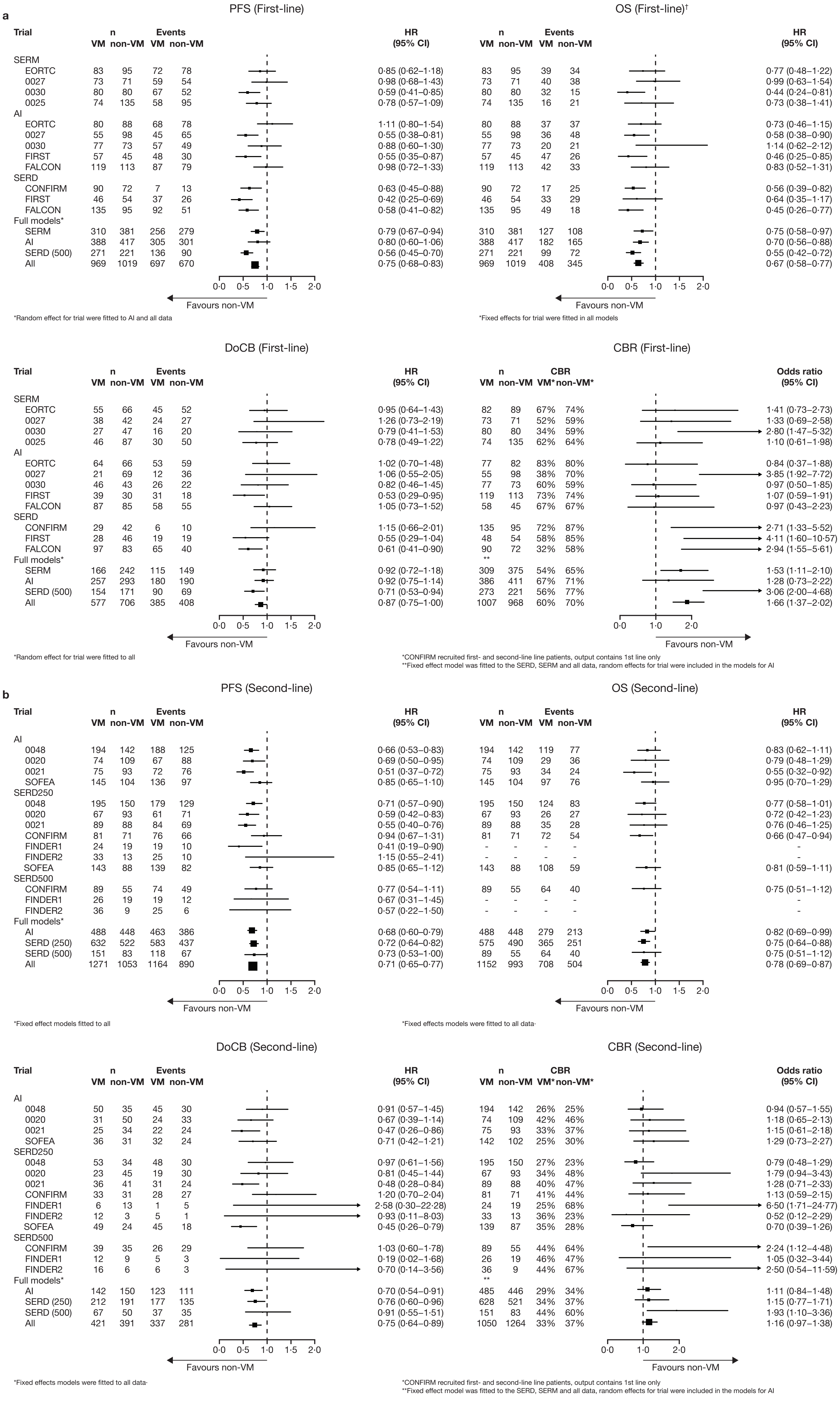
a
PFS (First-line)
Trial | n VM | n non-VM | Events VM | Events non-VM | HR (95% CI)
SERM
EORTC 83 95 72 78 0·85 (0·62–1·18)
0027 73 71 59 54 0·98 (0·68–1·43)
0030 80 80 67 52 0·59 (0·41–0·85)
0025 74 135 58 95 0·78 (0·57–1·09)
AI
EORTC 80 88 68 78 1·11 (0·80–1·54)
0027 55 98 45 65 0·55 (0·38–0·81)
0030 77 73 57 49 0·88 (0·60–1·30)
FIRST 57 45 48 30 0·55 (0·35–0·87)
FALCON 119 113 87 79 0·98 (0·72–1·33)
SERD
CONFIRM 90 72 7 13 0·63 (0·45–0·88)
FIRST 46 54 37 26 0·42 (0·25–0·69)
FALCON 135 95 92 51 0·58 (0·41–0·82)
Full models*
SERM 310 381 256 279 0·79 (0·67–0·94)
AI 388 417 305 301 0·80 (0·60–1·06)
SERD (500) 271 221 136 90 0·56 (0·45–0·70)
All 969 1019 697 670 0·75 (0·68–0·83)
0·0 0·5 1·0 1·5 2·0
Favours non-VM
*Random effect for trial were fitted to AI and all data
OS (First-line)†
n VM | n non-VM | Events VM | Events non-VM | HR (95% CI)
83 95 39 34 0·77 (0·48–1·22)
73 71 40 38 0·99 (0·63–1·54)
80 80 32 15 0·44 (0·24–0·81)
74 135 16 21 0·73 (0·38–1·41)
80 88 37 37 0·73 (0·46–1·15)
55 98 36 48 0·58 (0·38–0·90)
77 73 20 21 1·14 (0·62–2·12)
57 45 47 26 0·46 (0·25–0·85)
119 113 42 33 0·83 (0·52–1·31)
90 72 17 25 0·56 (0·39–0·82)
46 54 33 29 0·64 (0·35–1·17)
135 95 49 18 0·45 (0·26–0·77)
310 381 127 108 0·75 (0·58–0·97)
388 417 182 165 0·70 (0·56–0·88)
271 221 99 72 0·55 (0·42–0·72)
969 1019 408 345 0·67 (0·58–0·77)
0·0 0·5 1·0 1·5 2·0
Favours non-VM
*Fixed effects for trial were fitted in all models
DoCB (First-line)
Trial | n VM | n non-VM | Events VM | Events non-VM | HR (95% CI)
SERM
EORTC 55 66 45 52 0·95 (0·64–1·43)
0027 38 42 24 27 1·26 (0·73–2·19)
0030 27 47 16 20 0·79 (0·41–1·53)
0025 46 87 30 50 0·78 (0·49–1·22)
AI
EORTC 64 66 53 59 1·02 (0·70–1·48)
0027 21 69 12 36 1·06 (0·55–2·05)
0030 46 43 26 22 0·82 (0·46–1·45)
FIRST 39 30 31 18 0·53 (0·29–0·95)
FALCON 87 85 58 55 1·05 (0·73–1·52)
SERD
CONFIRM 29 42 6 10 1·15 (0·66–2·01)
FIRST 28 46 19 19 0·55 (0·29–1·04)
FALCON 97 83 65 40 0·61 (0·41–0·90)
Full models*
SERM 166 242 115 149 0·92 (0·72–1·18)
AI 257 293 180 190 0·92 (0·75–1·14)
SERD (500) 154 171 90 69 0·71 (0·53–0·94)
All 577 706 385 408 0·87 (0·75–1·00)
0·0 0·5 1·0 1·5 2·0
Favours non-VM
*Random effect for trial were fitted to all
CBR (First-line)
n VM | n non-VM | CBR VM* | CBR non-VM* | Odds ratio (95% CI)
82 89 67% 74% 1·41 (0·73–2·73)
73 71 52% 59% 1·33 (0·69–2·58)
80 80 34% 59% 2·80 (1·47–5·32)
74 135 62% 64% 1·10 (0·61–1·98)
77 82 83% 80% 0·84 (0·37–1·88)
55 98 38% 70% 3·85 (1·92–7·72)
77 73 60% 59% 0·97 (0·50–1·85)
119 113 73% 74% 1·07 (0·59–1·91)
58 45 67% 67% 0·97 (0·43–2·23)
135 95 72% 87% 2·71 (1·33–5·52)
48 54 58% 85% 4·11 (1·60–10·57)
90 72 32% 58% 2·94 (1·55–5·61)
**
309 375 54% 65% 1·53 (1·11–2·10)
386 411 67% 71% 1·28 (0·73–2·22)
273 221 56% 77% 3·06 (2·00–4·68)
1007 968 60% 70% 1·66 (1·37–2·02)
0·0 1·0 2·0
Favours non-VM
*CONFIRM recruited first- and second-line line patients, output contains 1st line only
**Fixed effect model was fitted to the SERD, SERM and all data, random effects for trial were included in the models for AI
b
PFS (Second-line)
Trial | n VM | n non-VM | Events VM | Events non-VM | HR (95% CI)
AI
0048 194 142 188 125 0·66 (0·53–0·83)
0020 74 109 67 88 0·69 (0·50–0·95)
0021 75 93 72 76 0·51 (0·37–0·72)
SOFEA 145 104 136 97 0·85 (0·65–1·10)
SERD250
0048 195 150 179 129 0·71 (0·57–0·90)
0020 67 93 61 71 0·59 (0·42–0·83)
0021 89 88 84 69 0·55 (0·40–0·76)
CONFIRM 81 71 76 66 0·94 (0·67–1·31)
FINDER1 24 19 19 10 0·41 (0·19–0·90)
FINDER2 33 13 25 10 1·15 (0·55–2·41)
SOFEA 143 88 139 82 0·85 (0·65–1·12)
SERD500
CONFIRM 89 55 74 49 0·77 (0·54–1·11)
FINDER1 26 19 19 12 0·67 (0·31–1·45)
FINDER2 36 9 25 6 0·57 (0·22–1·50)
Full models*
AI 488 448 463 386 0·68 (0·60–0·79)
SERD (250) 632 522 583 437 0·72 (0·64–0·82)
SERD (500) 151 83 118 67 0·73 (0·53–1·00)
All 1271 1053 1164 890 0·71 (0·65–0·77)
0·0 0·5 1·0 1·5 2·0
Favours non-VM
*Fixed effect models fitted to all
OS (Second-line)
n VM | n non-VM | Events VM | Events non-VM | HR (95% CI)
194 142 119 77 0·83 (0·62–1·11)
74 109 29 36 0·79 (0·48–1·29)
75 93 34 24 0·55 (0·32–0·92)
145 104 97 76 0·95 (0·70–1·29)
195 150 124 83 0·77 (0·58–1·01)
67 93 26 27 0·72 (0·42–1·23)
89 88 35 28 0·76 (0·46–1·25)
81 71 72 54 0·66 (0·47–0·94)
- - - - -
- - - - -
143 88 108 59 0·81 (0·59–1·11)
89 55 64 40 0·75 (0·51–1·12)
- - - - -
- - - - -
488 448 279 213 0·82 (0·69–0·99)
575 490 365 251 0·75 (0·64–0·88)
89 55 64 40 0·75 (0·51–1·12)
1152 993 708 504 0·78 (0·69–0·87)
0·0 0·5 1·0 1·5 2·0
Favours non-VM
*Fixed effects models were fitted to all data·
DoCB (Second-line)
Trial | n VM | n non-VM | Events VM | Events non-VM | HR (95% CI)
AI
0048 50 35 45 30 0·91 (0·57–1·45)
0020 31 50 24 33 0·67 (0·39–1·14)
0021 25 34 22 24 0·47 (0·26–0·86)
SOFEA 36 31 32 24 0·71 (0·42–1·21)
SERD250
0048 53 34 48 30 0·97 (0·61–1·56)
0020 23 45 19 30 0·81 (0·45–1·44)
0021 36 41 31 24 0·48 (0·28–0·84)
CONFIRM 33 31 28 27 1·20 (0·70–2·04)
FINDER1 6 13 1 5 2·58 (0·30–22·28)
FINDER2 12 3 5 1 0·93 (0·11–8·03)
SOFEA 49 24 45 18 0·45 (0·26–0·79)
SERD500
CONFIRM 39 35 26 29 1·03 (0·60–1·78)
FINDER1 12 9 5 3 0·19 (0·02–1·68)
FINDER2 16 6 6 3 0·70 (0·14–3·56)
Full models*
AI 142 150 123 111 0·70 (0·54–0·91)
SERD (250) 212 191 177 135 0·76 (0·60–0·96)
SERD (500) 67 50 37 35 0·91 (0·55–1·51)
All 421 391 337 281 0·75 (0·64–0·89)
0·0 0·5 1·0 1·5 2·0
Favours non-VM
*Fixed effects models were fitted to all data·
CBR (Second-line)
n VM | n non-VM | CBR VM* | CBR non-VM* | Odds ratio (95% CI)
194 142 26% 25% 0·94 (0·57–1·55)
74 109 42% 46% 1·18 (0·65–2·13)
75 93 33% 37% 1·15 (0·61–2·18)
142 102 25% 30% 1·29 (0·73–2·27)
195 150 27% 23% 0·79 (0·48–1·29)
67 93 34% 48% 1·79 (0·94–3·43)
89 88 40% 47% 1·28 (0·71–2·33)
81 71 41% 44% 1·13 (0·59–2·15)
24 19 25% 68% 6·50 (1·71–24·77)
33 13 36% 23% 0·52 (0·12–2·29)
139 87 35% 28% 0·70 (0·39–1·26)
89 55 44% 64% 2·24 (1·12–4·48)
26 19 46% 47% 1·05 (0·32–3·44)
36 9 44% 67% 2·50 (0·54–11·59)
**
485 446 29% 34% 1·11 (0·84–1·48)
628 521 34% 37% 1·15 (0·77–1·71)
151 83 44% 60% 1·93 (1·10–3·36)
1050 1264 33% 37% 1·16 (0·97–1·38)
0·0 1·0 2·0
Favours non-VM
*CONFIRM recruited first- and second-line line patients, output contains 1st line only
**Fixed effect model was fitted to the SERD, SERM and all data, random effects for trial were included in the models for AI

Supplementary Figure 1: Clinical outcomes for VM versus non-VM by endocrine agent and study

a Forest plots of PFS, OS, DoCB and CBR in the first-line setting. **b** Forest plots of PFS, OS, DoCB and CBR in the second-line setting.

AI, aromatase inhibitor; CBR, clinical benefit rate; DoCB, duration of clinical benefit; HR, hazard ratio; n, number of patients; non-VM, non-visceral metastases; PFS, progression-free survival; OR, odds ratio; OS, overall survival; SERD, selective estrogen receptor degrader; SERM, selective estrogen receptor modulator; VLM, visceral liver metastases; VM, visceral metastases; VnLM, visceral non-liver metastases.

a

PFS (First-line)

Trial	n VLM	n VnLM	Events VLM	Events VnLM	HR (95% CI)
SERM					
0027	15	58	12	47	0·60 (0·31–1·15)
0030	26	54	23	44	0·60 (0·36–0·99)
0025	23	51	20	38	0·70 (0·40–1·20)
AI					
0027	24	31	21	24	0·60 (0·33–1·08)
0030	13	64	9	48	0·83 (0·40–1·71)
FIRST	13	44	11	37	0·83 (0·42–1·62)
FALCON	41	78	32	55	0·62 (0·40–0·96)
SERD (500)					
CONFIRM	47	43	46	37	0·54 (0·34–0·85)
FIRST	14	32	14	23	0·61 (0·31–1·20)
FALCON	38	97	30	62	0·57 (0·37–0·89)
Full models*					
SERM	64	163	55	129	0·63 (0·46–0·87)
AI	91	217	73	164	0·68 (0·51–0·90)
SERD	99	172	90	122	0·56 (0·42–0·75)
All	254	552	218	415	0·62 (0·52–0·74)

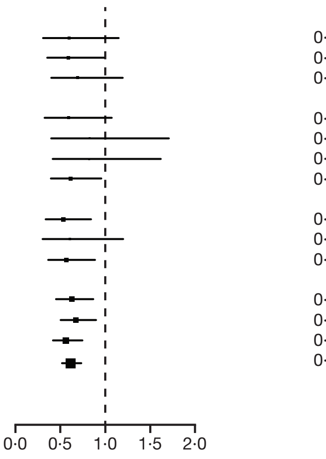

*Fixed effect for trial were fitted in all models

OS (First-line)

Trial	n VLM	n VnLM	Events VLM	Events VnLM	HR (95% CI)
SERM					
0027	15	58	10	30	0·56 (0·27–1·14)
0030	26	54	12	20	0·54 (0·26–1·12)
0025	23	51	6	10	0·72 (0·26–2·00)
AI					
0027	24	31	21	15	0·35 (0·18–0·68)
0030	13	64	4	16	0·52 (0·17–1·61)
FIRST	13	44	10	37	1·39 (0·68–2·84)
FALCON	41	78	19	23	0·51 (0·28–0·94)
SERD (500)					
CONFIRM	47	43	42	31	0·52 (0·32–0·83)
FIRST	14	32	13	20	0·64 (0·32–1·29)
FALCON	38	97	23	26	0·34 (0·19–0·60)
Full models*					
SERM	64	163	28	60	0·58 (0·37–0·92)
AI	91	217	54	91	0·60 (0·32–1·12)
SERD	99	172	78	77	0·47 (0·34–0·66)
All	254	552	160	228	0·54 (0·43–0·66)

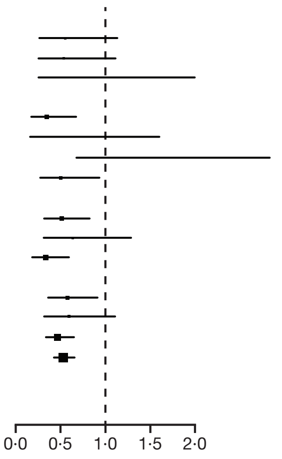

*Random effect for trial were fitted in model for AI

DoCB (First-line)

Trial	n VLM	n VnLM	Events VLM	Events VnLM	HR (95% CI)
SERM					
0027	5	33	2	22	0·87 (0·19–3·84)
0030	7	20	5	11	0·61 (0·21–1·77)
0025	13	33	10	20	0·74 (0·35–1·59)
AI					
0027	6	15	4	8	0·80 (0·24–2·71)
0030	7	39	3	23	0·84 (0·25–2·90)
FIRST	9	30	7	24	0·79 (0·34–1·83)
FALCON	24	63	17	41	0·75 (0·42–1·32)
SERD (500)					
CONFIRM	13	16	13	10	0·22 (0·08–0·57)
FIRST	8	20	8	11	0·36 (0·14–0·93)
FALCON	21	76	17	48	0·72 (0·41–1·25)
Full models*					
SERM	25	86	17	53	0·72 (0·40–1·27)
AI	46	147	31	96	0·77 (0·51–1·17)
SERD	42	112	38	69	0·49 (0·32–0·76)
All	113	345	86	218	0·64 (0·49–0·84)

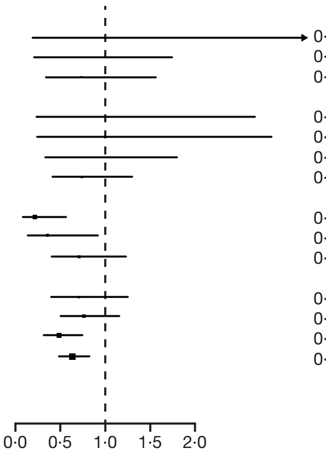

*Fixed effect for trial were fitted in all models

CBR (First-line)

Trial	n VLM	n VnLM	CBR VLM*	CBR VnLM*	Odds ratio (95% CI)
SERM					
0027	15	58	33%	57%	2·64 (0·80–8·70)
0030	26	54	27%	37%	1·60 (0·57–4·46)
0025	23	51	57%	65%	1·41 (0·52–3·85)
AI					
0027	24	31	25%	48%	2·81 (0·88–8·99)
0030	13	64	54%	61%	1·34 (0·40–4·44)
FIRST	12	44	69%	68%	0·95 (0·25–3·63)
FALCON	41	78	59%	81%	2·98 (1·29–6·88)
SERD (500)					
CONFIRM	47	43	28%	37%	1·55 (0·64–3·77)
FIRST	14	32	57%	62%	1·25 (0·35–4·49)
FALCON	38	97	55%	78%	2·93 (1·31–6·53)
Full models*					
SERM	64	163	39%	53%	1·74 (0·94–3·22)
AI	91	217	51%	68%	2·07 (1·21–3·56)
SERD	99	172	42%	65%	1·99 (1·16–3·41)
All	552	254	44%	62%	1·95 (1·41–2·69)

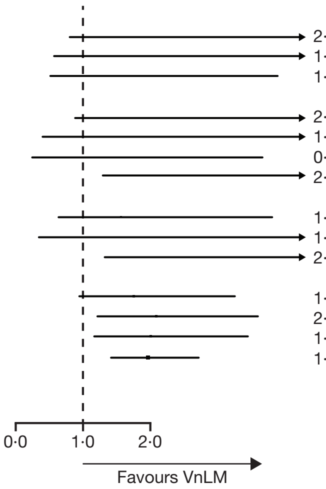

*Fixed effect for trial were fitted in all models

b

PFS (Second-line)

Trial	n VLM	n VnLM	Events VLM	Events VnLM	HR (95% CI)
AI					
0048	106	88	103	85	0·74 (0·55–0·99)
0020	42	32	40	27	0·62 (0·38–1·02)
0021	38	37	37	35	0·68 (0·43–1·09)
SOFEA	72	73	70	66	0·43 (0·30–0·61)
SERD250					
0048	109	86	100	79	0·73 (0·54–0·98)
0020	39	28	37	24	0·58 (0·34–0·99)
0021	44	45	44	40	0·63 (0·40–0·99)
CONFIRM	41	40	40	36	0·59 (0·37–0·94)
FINDER1	9	15	8	11	0·50 (0·19–1·29)
FINDER2	19	14	15	10	0·84 (0·38–1·87)
SOFEA	72	71	71	68	0·59 (0·42–0·83)
SERD500					
CONFIRM	36	53	31	43	0·83 (0·52–1·32)
FINDER1	9	17	6	13	1·10 (0·41–2·94)
FINDER2	19	17	17	8	0·17 (0·06–0·47)
Full models*					
AI	258	230	250	213	0·61 (0·51–0·74)
SERD (250)	333	299	315	268	0·64 (0·54–0·76)
SERD (500)	64	87	54	64	0·56 (0·19–1·64)
All	655	616	619	545	0·63 (0·56–0·71)

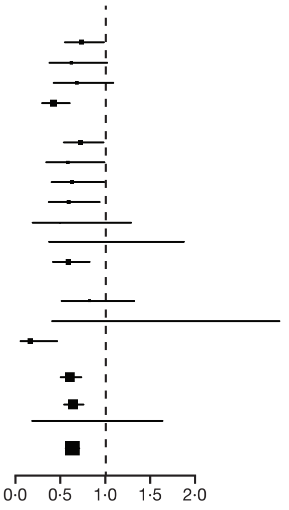

*Random effects for trial were fitted in SERD (500) model.

OS (Second-line)

Trial	n VLM	n VnLM	Events VLM	Events VnLM	HR (95% CI)
AI					
0048	106	88	75	44	0·49 (0·34–0·71)
0020	42	32	22	7	0·32 (0·13–0·75)
0021	38	37	21	13	0·37 (0·17–0·81)
SOFEA	72	73	57	40	0·48 (0·32–0·72)
SERD250					
0048	109	86	79	45	0·59 (0·41–0·86)
0020	39	28	17	9	0·62 (0·28–1·39)
0021	44	45	21	14	0·54 (0·27–1·07)
CONFIRM	41	40	39	33	0·60 (0·38–0·96)
FINDER1	-	-	-	-	-
FINDER2	-	-	-	-	-
SOFEA	72	71	59	49	0·71 (0·49–1·05)
SERD500					
CONFIRM	36	53	27	37	0·73 (0·44–1·20)
FINDER1	-	-	-	-	-
FINDER2	-	-	-	-	-
Full models*					
AI	258	230	175	104	0·45 (0·35–0·58)
SERD (250)	305	270	215	150	0·63 (0·51–0·77)
SERD (500)	36	53	27	37	0·73 (0·44–1·20)
All	599	553	417	291	0·56 (0·48–0·65)

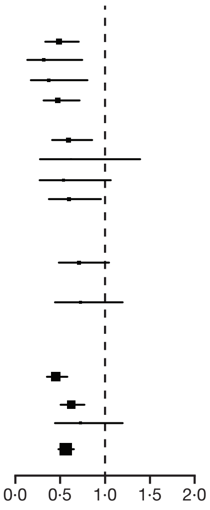

*Fixed effect models fitted to all

DoCB (Second-line)

Trial	n VLM	n non-VM	Events VLM	Events VnLM	HR (95% CI)
AI					
0048	21	29	18	27	1·00 (0·54–1·83)
0020	14	17	12	12	0·73 (0·33–1·65)
0021	10	15	9	13	0·98 (0·41–2·33)
SOFEA	10	26	10	22	0·55 (0·25–1·22)
SERD250					
0048	20	33	18	30	0·73 (0·54–0·98)
0020	9	14	8	11	0·58 (0·34–0·99)
0021	14	22	14	17	0·63 (0·40–0·99)
CONFIRM	13	20	12	16	0·59 (0·37–0·94)
FINDER1	-	-	-	-	-
FINDER2	6	6	3	2	0·84 (0·38–1·87)
SOFEA	18	31	17	28	0·59 (0·42–0·83)
SERD500					
CONFIRM	11	28	7	19	0·83 (0·52–1·32)
FINDER1	-	-	-	-	-
FINDER2	4	12	2	4	0·17 (0·06–0·47)
Full models*					
AI	55	87	49	74	0·81 (0·56–1·18)
SERD (250)	80	126	72	104	0·65 (0·55–0·77)
SERD (500)	15	40	9	23	0·40 (0·08–1·90)
All	150	253	130	201	0·67 (0·58–0·77)

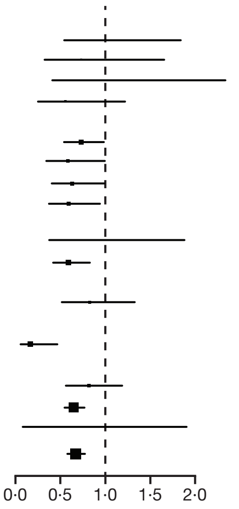

*Random effects for trial were fitted in model for SERD (500).

CBR (Second-line)

Trial	n VLM	n VnLM	CBR VLM*	CBR VnLM*	Odds ratio (95% CI)
AI					
0048	106	88	20%	33%	1·99 (1·04–3·82)
0020	42	32	33%	53%	2·27 (0·88–5·83)
0021	38	37	26%	41%	1·91 (0·72–5·07)
SOFEA	71	71	14%	37%	3·52 (1·54–8·04)
SERD250					
0048	109	86	18%	38%	2·77 (1·44–5·31)
0020	39	28	23%	50%	3·33 (1·17–9·53)
0021	44	45	32%	49%	2·05 (0·87–4·86)
CONFIRM	41	40	32%	50%	2·15 (0·87–5·32)
FINDER1	9	15	11%	33%	4·00 (0·39–41·51)
FINDER2	19	14	32%	43%	1·63 (0·39–6·82)
SOFEA	70	69	26%	45%	2·36 (1·15–4·82)
SERD500					
CONFIRM	36	53	31%	53%	2·55 (1·04–6·20)
FINDER1	9	17	44%	47%	1·11 (0·22–5·63)
FINDER2	19	17	21%	71%	9·00 (1·97–41·08)
Full models*	**				
AI	257	228	21%	38%	2·33 (1·55–3·50)
SERD (250)	331	297	24%	44%	2·45 (1·73–3·45)
SERD (500)	64	87	30%	55%	2·85 (1·42–5·71)
All	612	652	24%	43%	2·45 (1·91–3·13)

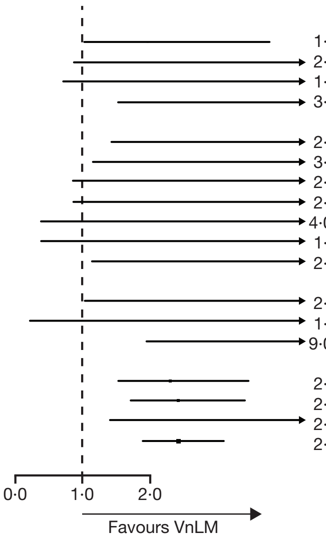

*Fixed effect model was fitted to all models

Supplementary Figure 2: Clinical outcomes for VnLM and VLM by endocrine agent and study

a Forest plots of PFS, OS, DoCB and CBR in the first-line setting. **b** Forest plots of PFS, OS, DoCB and CBR second-line setting.

AI, aromatase inhibitor; CBR, clinical benefit rate; DoCB, duration of clinical benefit; HR, hazard ratio; n, number of patients; non-VM, non-visceral metastases; PFS, progression-free survival; OR, odds ratio; OS, overall survival; SERD, selective estrogen receptor degrader; SERM, selective estrogen receptor modulator; VLM, visceral liver metastases; VM, visceral metastases; VnLM, visceral non-liver metastases.

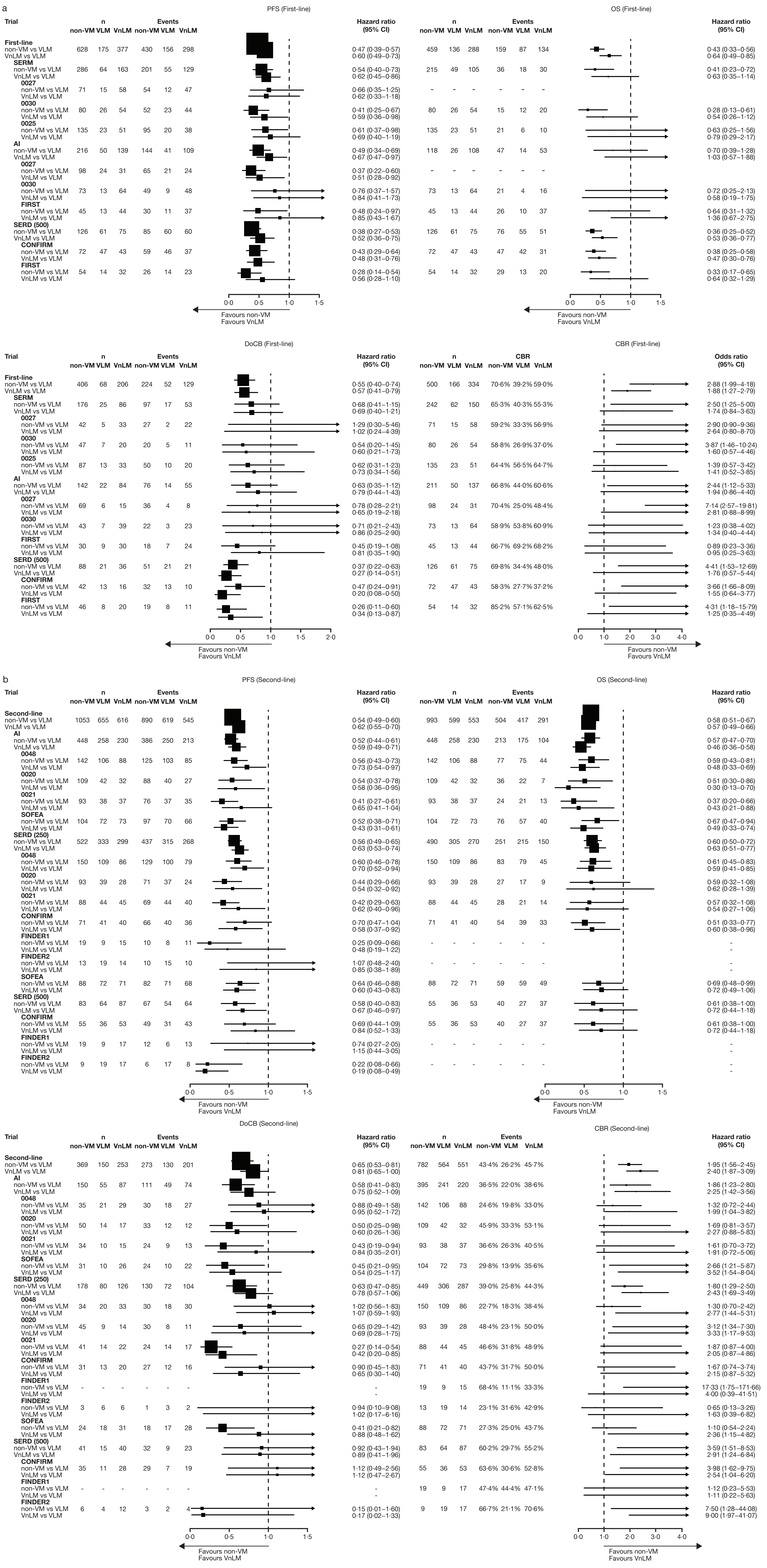
a
PFS (First-line)
Trial | n: non-VM VLM VnLM | Events: non-VM VLM VnLM | Hazard ratio (95% CI)
First-line
non-VM vs VLM 628 175 377 430 156 298 0·47 (0·39–0·57)
VnLM vs VLM 0·60 (0·49–0·73)
SERM
non-VM vs VLM 286 64 163 201 55 129 0·54 (0·40–0·73)
VnLM vs VLM 0·62 (0·45–0·86)
0027
non-VM vs VLM 71 15 58 54 12 47 0·66 (0·35–1·25)
VnLM vs VLM 0·62 (0·33–1·18)
0030
non-VM vs VLM 80 26 54 52 23 44 0·41 (0·25–0·67)
VnLM vs VLM 0·59 (0·36–0·98)
0025
non-VM vs VLM 135 23 51 95 20 38 0·61 (0·37–0·98)
VnLM vs VLM 0·69 (0·40–1·19)
AI
non-VM vs VLM 216 50 139 144 41 109 0·49 (0·34–0·69)
VnLM vs VLM 0·67 (0·47–0·97)
0027
non-VM vs VLM 98 24 31 65 21 24 0·37 (0·22–0·60)
VnLM vs VLM 0·51 (0·28–0·92)
0030
non-VM vs VLM 73 13 64 49 9 48 0·76 (0·37–1·57)
VnLM vs VLM 0·84 (0·41–1·73)
FIRST
non-VM vs VLM 45 13 44 30 11 37 0·48 (0·24–0·97)
VnLM vs VLM 0·85 (0·43–1·67)
SERD (500)
non-VM vs VLM 126 61 75 85 60 60 0·38 (0·27–0·53)
VnLM vs VLM 0·52 (0·36–0·75)
CONFIRM
non-VM vs VLM 72 47 43 59 46 37 0·43 (0·29–0·64)
VnLM vs VLM 0·48 (0·31–0·76)
FIRST
non-VM vs VLM 54 14 32 26 14 23 0·28 (0·14–0·54)
VnLM vs VLM 0·56 (0·28–1·10)
0·0 0·5 1·0 1·5
Favours non-VM
Favours VnLM
OS (First-line)
n: non-VM VLM VnLM | Events: non-VM VLM VnLM | Hazard ratio (95% CI)
First-line: 459 136 288 159 87 134 0·43 (0·33–0·56) / 0·64 (0·49–0·85)
SERM: 215 49 105 36 18 30 0·41 (0·23–0·72) / 0·63 (0·35–1·14)
0027: - - - - - -
0030: 80 26 54 15 12 20 0·28 (0·13–0·61) / 0·54 (0·26–1·12)
0025: 135 23 51 21 6 10 0·63 (0·25–1·56) / 0·79 (0·29–2·17)
AI: 118 26 108 47 14 53 0·70 (0·39–1·28) / 1·03 (0·57–1·88)
0027: - - - - - -
0030: 73 13 64 21 4 16 0·72 (0·25–2·13) / 0·58 (0·19–1·75)
FIRST: 45 13 44 26 10 37 0·64 (0·31–1·32) / 1·36 (0·67–2·75)
SERD (500): 126 61 75 76 55 51 0·36 (0·25–0·52) / 0·53 (0·36–0·77)
CONFIRM: 72 47 43 47 42 31 0·38 (0·25–0·58) / 0·47 (0·30–0·76)
FIRST: 54 14 32 29 13 20 0·33 (0·17–0·65) / 0·64 (0·32–1·29)
0·0 0·5 1·0 1·5
Favours non-VM
Favours VnLM
DoCB (First-line)
Trial | n: non-VM VLM VnLM | Events: non-VM VLM VnLM | Hazard ratio (95% CI)
First-line
non-VM vs VLM 406 68 206 224 52 129 0·55 (0·40–0·74)
VnLM vs VLM 0·57 (0·41–0·79)
SERM
non-VM vs VLM 176 25 86 97 17 53 0·68 (0·41–1·15)
VnLM vs VLM 0·69 (0·40–1·21)
0027
non-VM vs VLM 42 5 33 27 2 22 1·29 (0·30–5·46)
VnLM vs VLM 1·02 (0·24–4·39)
0030
non-VM vs VLM 47 7 20 20 5 11 0·54 (0·20–1·45)
VnLM vs VLM 0·60 (0·21–1·73)
0025
non-VM vs VLM 87 13 33 50 10 20 0·62 (0·31–1·23)
VnLM vs VLM 0·73 (0·34–1·56)
AI
non-VM vs VLM 142 22 84 76 14 55 0·63 (0·35–1·12)
VnLM vs VLM 0·79 (0·44–1·43)
0027
non-VM vs VLM 69 6 15 36 4 8 0·78 (0·28–2·21)
VnLM vs VLM 0·65 (0·19–2·18)
0030
non-VM vs VLM 43 7 39 22 3 23 0·71 (0·21–2·43)
VnLM vs VLM 0·86 (0·25–2·90)
FIRST
non-VM vs VLM 30 9 30 18 7 24 0·45 (0·19–1·08)
VnLM vs VLM 0·81 (0·35–1·90)
SERD (500)
non-VM vs VLM 88 21 36 51 21 21 0·37 (0·22–0·63)
VnLM vs VLM 0·27 (0·14–0·51)
CONFIRM
non-VM vs VLM 42 13 16 32 13 10 0·47 (0·24–0·91)
VnLM vs VLM 0·20 (0·08–0·50)
FIRST
non-VM vs VLM 46 8 20 19 8 11 0·26 (0·11–0·60)
VnLM vs VLM 0·34 (0·13–0·87)
0·0 0·5 1·0 1·5 2·0
Favours non-VM
Favours VnLM
CBR (First-line)
n: non-VM VLM VnLM | CBR: non-VM VLM VnLM | Odds ratio (95% CI)
First-line: 500 166 334 70·6% 39·2% 59·0% 2·88 (1·99–4·18) / 1·88 (1·27–2·79)
SERM: 242 62 150 65·3% 40·3% 55·3% 2·50 (1·25–5·00) / 1·74 (0·84–3·63)
0027: 71 15 58 59·2% 33·3% 56·9% 2·90 (0·90–9·36) / 2·64 (0·80–8·70)
0030: 80 26 54 58·8% 26·9% 37·0% 3·87 (1·46–10·24) / 1·60 (0·57–4·46)
0025: 135 23 51 64·4% 56·5% 64·7% 1·39 (0·57–3·42) / 1·41 (0·52–3·85)
AI: 211 50 137 66·8% 44·0% 60·6% 2·44 (1·12–5·33) / 1·94 (0·86–4·40)
0027: 98 24 31 70·4% 25·0% 48·4% 7·14 (2·57–19·81) / 2·81 (0·88–8·99)
0030: 73 13 64 58·9% 53·8% 60·9% 1·23 (0·38–4·02) / 1·34 (0·40–4·44)
FIRST: 45 13 44 66·7% 69·2% 68·2% 0·89 (0·23–3·36) / 0·95 (0·25–3·63)
SERD (500): 126 61 75 69·8% 34·4% 48·0% 4·41 (1·53–12·69) / 1·76 (0·57–5·44)
CONFIRM: 72 47 43 58·3% 27·7% 37·2% 3·66 (1·66–8·09) / 1·55 (0·64–3·77)
FIRST: 54 14 32 85·2% 57·1% 62·5% 4·31 (1·18–15·79) / 1·25 (0·35–4·49)
0·0 1·0 2·0 3·0 4·0
Favours non-VM
Favours VnLM
b
PFS (Second-line)
Trial | n: non-VM VLM VnLM | Events: non-VM VLM VnLM | Hazard ratio (95% CI)
Second-line
non-VM vs VLM 1053 655 616 890 619 545 0·54 (0·49–0·60)
VnLM vs VLM 0·62 (0·55–0·70)
AI
non-VM vs VLM 448 258 230 386 250 213 0·52 (0·44–0·61)
VnLM vs VLM 0·59 (0·49–0·71)
0048
non-VM vs VLM 142 106 88 125 103 85 0·56 (0·43–0·73)
VnLM vs VLM 0·73 (0·54–0·97)
0020
non-VM vs VLM 109 42 32 88 40 27 0·54 (0·37–0·78)
VnLM vs VLM 0·58 (0·36–0·95)
0021
non-VM vs VLM 93 38 37 76 37 35 0·41 (0·27–0·61)
VnLM vs VLM 0·65 (0·41–1·04)
SOFEA
non-VM vs VLM 104 72 73 97 70 66 0·52 (0·38–0·71)
VnLM vs VLM 0·43 (0·31–0·61)
SERD (250)
non-VM vs VLM 522 333 299 437 315 268 0·56 (0·49–0·65)
VnLM vs VLM 0·63 (0·53–0·74)
0048
non-VM vs VLM 150 109 86 129 100 79 0·60 (0·46–0·78)
VnLM vs VLM 0·70 (0·52–0·94)
0020
non-VM vs VLM 93 39 28 71 37 24 0·44 (0·29–0·66)
VnLM vs VLM 0·54 (0·32–0·92)
0021
non-VM vs VLM 88 44 45 69 44 40 0·42 (0·29–0·63)
VnLM vs VLM 0·62 (0·40–0·96)
CONFIRM
non-VM vs VLM 71 41 40 66 40 36 0·70 (0·47–1·04)
VnLM vs VLM 0·58 (0·37–0·92)
FINDER1
non-VM vs VLM 19 9 15 10 8 11 0·25 (0·09–0·66)
VnLM vs VLM 0·48 (0·19–1·22)
FINDER2
non-VM vs VLM 13 19 14 10 15 10 1·07 (0·48–2·40)
VnLM vs VLM 0·85 (0·38–1·89)
SOFEA
non-VM vs VLM 88 72 71 82 71 68 0·64 (0·46–0·88)
VnLM vs VLM 0·60 (0·43–0·83)
SERD (500)
non-VM vs VLM 83 64 87 67 54 64 0·58 (0·40–0·83)
VnLM vs VLM 0·67 (0·46–0·97)
CONFIRM
non-VM vs VLM 55 36 53 49 31 43 0·69 (0·44–1·09)
VnLM vs VLM 0·84 (0·52–1·33)
FINDER1
non-VM vs VLM 19 9 17 12 6 13 0·74 (0·27–2·05)
VnLM vs VLM 1·15 (0·44–3·05)
FINDER2
non-VM vs VLM 9 19 17 6 17 8 0·22 (0·08–0·66)
VnLM vs VLM 0·19 (0·08–0·49)
0·0 0·5 1·0 1·5
Favours non-VM
Favours VnLM
OS (Second-line)
n: non-VM VLM VnLM | Events: non-VM VLM VnLM | Hazard ratio (95% CI)
Second-line: 993 599 553 504 417 291 0·58 (0·51–0·67) / 0·57 (0·49–0·66)
AI: 448 258 230 213 175 104 0·57 (0·47–0·70) / 0·46 (0·36–0·58)
0048: 142 106 88 77 75 44 0·59 (0·43–0·81) / 0·48 (0·33–0·69)
0020: 109 42 32 36 22 7 0·51 (0·30–0·86) / 0·30 (0·13–0·70)
0021: 93 38 37 24 21 13 0·37 (0·20–0·66) / 0·43 (0·21–0·88)
SOFEA: 104 72 73 76 57 40 0·67 (0·47–0·94) / 0·49 (0·33–0·74)
SERD (250): 490 305 270 251 215 150 0·60 (0·50–0·72) / 0·63 (0·51–0·77)
0048: 150 109 86 83 79 45 0·61 (0·45–0·83) / 0·59 (0·41–0·85)
0020: 93 39 28 27 17 9 0·59 (0·32–1·08) / 0·62 (0·28–1·39)
0021: 88 44 45 28 21 14 0·57 (0·32–1·08) / 0·54 (0·27–1·06)
CONFIRM: 71 41 40 54 39 33 0·51 (0·33–0·77) / 0·60 (0·38–0·96)
FINDER1: - - - - - - - / -
FINDER2: - - - - - - - / -
SOFEA: 88 72 71 59 59 49 0·69 (0·48–0·99) / 0·72 (0·49–1·06)
SERD (500): 55 36 53 40 27 37 0·61 (0·38–1·00) / 0·72 (0·44–1·18)
CONFIRM: 55 36 53 40 27 37 0·61 (0·38–1·00) / 0·72 (0·44–1·18)
FINDER1: - - - - - - - / -
FINDER2: - - - - - - - / -
0·0 0·5 1·0 1·5
Favours non-VM
Favours VnLM
DoCB (Second-line)
Trial | n: non-VM VLM VnLM | Events: non-VM VLM VnLM | Hazard ratio (95% CI)
Second-line
non-VM vs VLM 369 150 253 273 130 201 0·65 (0·53–0·81)
VnLM vs VLM 0·81 (0·65–1·00)
AI
non-VM vs VLM 150 55 87 111 49 74 0·58 (0·41–0·83)
VnLM vs VLM 0·75 (0·52–1·09)
0048
non-VM vs VLM 35 21 29 30 18 27 0·88 (0·49–1·58)
VnLM vs VLM 0·95 (0·52–1·72)
0020
non-VM vs VLM 50 14 17 33 12 12 0·50 (0·25–0·98)
VnLM vs VLM 0·60 (0·26–1·36)
0021
non-VM vs VLM 34 10 15 24 9 13 0·43 (0·19–0·94)
VnLM vs VLM 0·84 (0·35–2·01)
SOFEA
non-VM vs VLM 31 10 26 24 10 22 0·45 (0·21–0·95)
VnLM vs VLM 0·54 (0·25–1·17)
SERD (250)
non-VM vs VLM 178 80 126 130 72 104 0·63 (0·47–0·85)
VnLM vs VLM 0·78 (0·57–1·06)
0048
non-VM vs VLM 34 20 33 30 18 30 1·02 (0·56–1·83)
VnLM vs VLM 1·07 (0·59–1·93)
0020
non-VM vs VLM 45 9 14 30 8 11 0·65 (0·29–1·42)
VnLM vs VLM 0·69 (0·28–1·75)
0021
non-VM vs VLM 41 14 22 24 14 17 0·27 (0·14–0·54)
VnLM vs VLM 0·42 (0·20–0·85)
CONFIRM
non-VM vs VLM 31 13 20 27 12 16 0·90 (0·45–1·83)
VnLM vs VLM 0·65 (0·30–1·40)
FINDER1
non-VM vs VLM - - - - - - -
VnLM vs VLM -
FINDER2
non-VM vs VLM 3 6 6 1 3 2 0·94 (0·10–9·08)
VnLM vs VLM 1·02 (0·17–6·16)
SOFEA
non-VM vs VLM 24 18 31 18 17 28 0·41 (0·21–0·82)
VnLM vs VLM 0·88 (0·48–1·62)
SERD (500)
non-VM vs VLM 41 15 40 32 9 23 0·92 (0·43–1·94)
VnLM vs VLM 0·89 (0·41–1·96)
CONFIRM
non-VM vs VLM 35 11 28 29 7 19 1·12 (0·49–2·56)
VnLM vs VLM 1·12 (0·47–2·67)
FINDER1
non-VM vs VLM - - - - - - -
VnLM vs VLM -
FINDER2
non-VM vs VLM 6 4 12 3 2 4 0·15 (0·01–1·60)
VnLM vs VLM 0·17 (0·02–1·33)
0·0 0·5 1·0 1·5
Favours non-VM
Favours VnLM
CBR (Second-line)
n: non-VM VLM VnLM | Events: non-VM VLM VnLM | Hazard ratio (95% CI)
Second-line: 782 564 551 43·4% 26·2% 45·7% 1·95 (1·56–2·45) / 2·40 (1·87–3·09)
AI: 395 241 220 36·5% 22·0% 38·6% 1·86 (1·23–2·80) / 2·25 (1·42–3·56)
0048: 142 106 88 24·6% 19·8% 33·0% 1·32 (0·72–2·44) / 1·99 (1·04–3·82)
0020: 109 42 32 45·9% 33·3% 53·1% 1·69 (0·81–3·57) / 2·27 (0·88–5·83)
0021: 93 38 37 36·6% 26·3% 40·5% 1·61 (0·70–3·72) / 1·91 (0·72–5·06)
SOFEA: 104 72 73 29·8% 13·9% 35·6% 2·66 (1·21–5·87) / 3·52 (1·54–8·04)
SERD (250): 449 306 287 39·0% 25·8% 44·3% 1·80 (1·29–2·50) / 2·43 (1·69–3·49)
0048: 150 109 86 22·7% 18·3% 38·4% 1·30 (0·70–2·42) / 2·77 (1·44–5·31)
0020: 93 39 28 48·4% 23·1% 50·0% 3·12 (1·34–7·30) / 3·33 (1·17–9·53)
0021: 88 44 45 46·6% 31·8% 48·9% 1·87 (0·87–4·00) / 2·05 (0·87–4·86)
CONFIRM: 71 41 40 43·7% 31·7% 50·0% 1·67 (0·74–3·74) / 2·15 (0·87–5·32)
FINDER1: 19 9 15 68·4% 11·1% 33·3% 17·33 (1·75–171·66) / 4·00 (0·39–41·51)
FINDER2: 13 19 14 23·1% 31·6% 42·9% 0·65 (0·13–3·26) / 1·63 (0·39–6·82)
SOFEA: 88 72 71 27·3% 25·0% 43·7% 1·10 (0·54–2·24) / 2·36 (1·15–4·82)
SERD (500): 83 64 87 60·2% 29·7% 55·2% 3·59 (1·51–8·53) / 2·91 (1·24–6·84)
CONFIRM: 55 36 53 63·6% 30·6% 52·8% 3·98 (1·62–9·75) / 2·54 (1·04–6·20)
FINDER1: 19 9 17 47·4% 44·4% 47·1% 1·12 (0·23–5·53) / 1·11 (0·22–5·63)
FINDER2: 9 19 17 66·7% 21·1% 70·6% 7·50 (1·28–44·08) / 9·00 (1·97–41·07)
0·0 1·0 2·0 3·0 4·0
Favours non-VM
Favours VnLM

Supplementary Figure 3: Clinical outcomes for non-VM and VnLM versus VLM by endocrine agent

a Forest plots of PFS, OS, DoCB and CBR in the first-line setting. **b** Forest plots of PFS, OS, DoCB and CBR in the second-line setting.

AI, aromatase inhibitor; CBR, clinical benefit rate; DoCB, duration of clinical benefit; HR, hazard ratio; n, number of patients; non-VM, non-visceral metastases; PFS, progression-free survival; OR, odds ratio; OS, overall survival; SERD, selective estrogen receptor degrader; SERM, selective estrogen receptor modulator; VLM, visceral liver metastases; VM, visceral metastases; VnLM, visceral non-liver metastases.

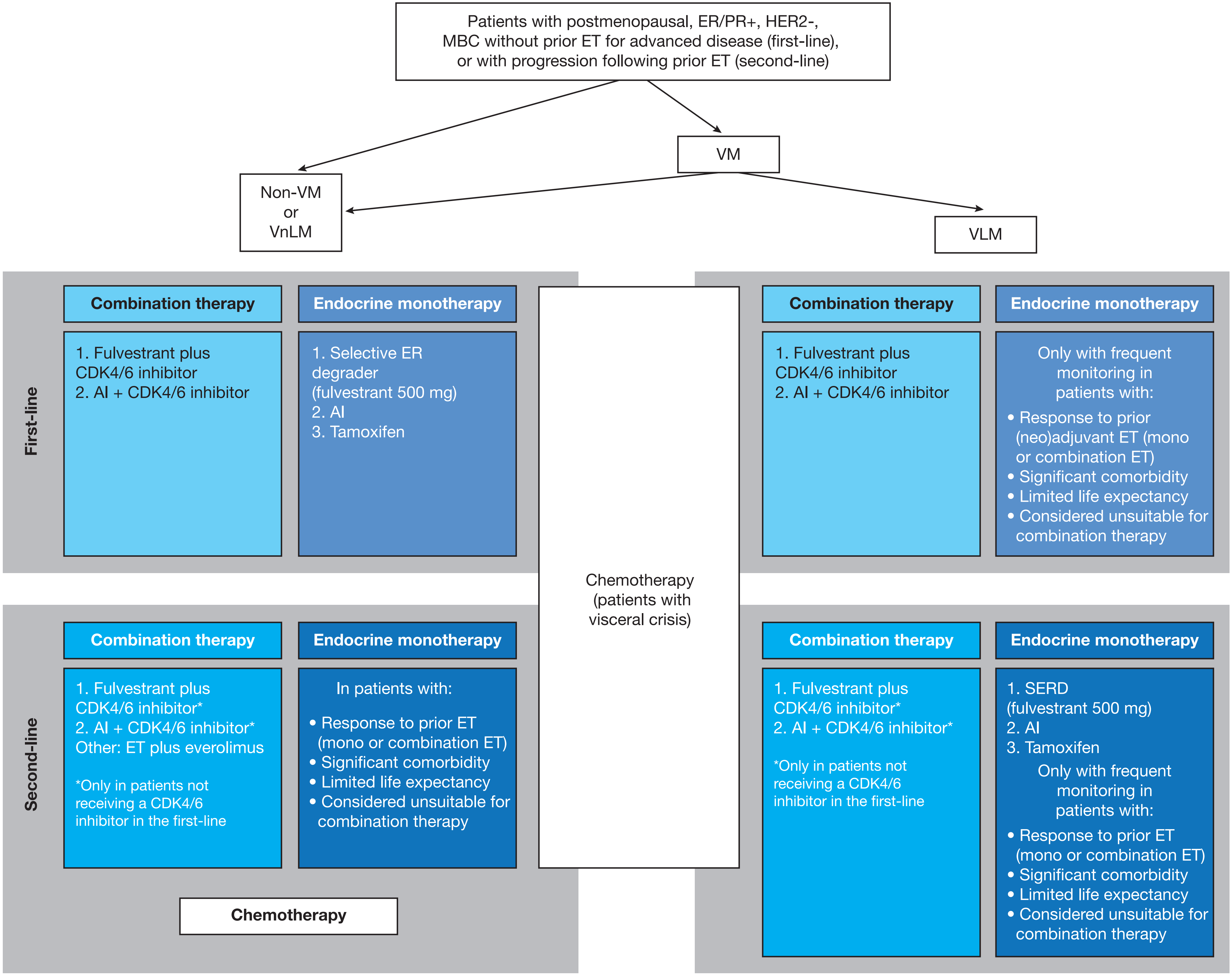
Patients with postmenopausal, ER/PR+, HER2-, MBC without prior ET for advanced disease (first-line), or with progression following prior ET (second-line)
VM
Non-VM or VnLM
VLM
First-line
Combination therapy
1. Fulvestrant plus CDK4/6 inhibitor
2. AI + CDK4/6 inhibitor
Endocrine monotherapy
1. Selective ER degrader (fulvestrant 500 mg)
2. AI
3. Tamoxifen
Chemotherapy (patients with visceral crisis)
Combination therapy
1. Fulvestrant plus CDK4/6 inhibitor
2. AI + CDK4/6 inhibitor
Endocrine monotherapy
Only with frequent monitoring in patients with:
• Response to prior (neo)adjuvant ET (mono or combination ET)
• Significant comorbidity
• Limited life expectancy
• Considered unsuitable for combination therapy
Second-line
Combination therapy
1. Fulvestrant plus CDK4/6 inhibitor*
2. AI + CDK4/6 inhibitor*
Other: ET plus everolimus
*Only in patients not receiving a CDK4/6 inhibitor in the first-line
Endocrine monotherapy
In patients with:
• Response to prior ET (mono or combination ET)
• Significant comorbidity
• Limited life expectancy
• Considered unsuitable for combination therapy
Chemotherapy
Combination therapy
1. Fulvestrant plus CDK4/6 inhibitor*
2. AI + CDK4/6 inhibitor*
*Only in patients not receiving a CDK4/6 inhibitor in the first-line
Endocrine monotherapy
1. SERD (fulvestrant 500 mg)
2. AI
3. Tamoxifen
Only with frequent monitoring in patients with:
• Response to prior ET (mono or combination ET)
• Significant comorbidity
• Limited life expectancy
• Considered unsuitable for combination therapy

Supplementary Figure 4: Potential first- and second-line treatment options in patients with HR+ ABC with or without VM

ABC, advanced breast cancer; AI, aromatase inhibitor; CDK, cyclin-dependent kinase; ER+, estrogen receptor-positive; ET, endocrine therapy; HER2-, human epidermal growth factor 2-negative; HR+, hormone receptor-positive; PR+, progesterone receptor-positive; SERD, selective estrogen receptor degrader; VLM, visceral liver metastases; VM, visceral metastases; VnLM, visceral non-liver metastases.

Supplementary References

1. Robertson, J.F.R. et al. Fulvestrant 500 mg versus anastrozole 1 mg for hormone receptor-positive advanced breast cancer (FALCON): an international, randomised, double-blind, phase 3 trial. *Lancet* **388**, 2997-3005 (2016)

2. Ellis, M.J. et al. Fulvestrant 500 mg versus anastrozole 1 mg for the first-line treatment of advanced breast cancer: overall survival analysis from the Phase II FIRST study. *J. Clin. Oncol.* **33**, 3781-3787 (2015).

3. Robertson, J.F.R. et al. Fulvestrant 500 mg versus anastrozole 1 mg for the first-line treatment of advanced breast cancer: follow-up analysis from the randomized 'FIRST' study. *Breast Cancer Res. Treat.* **136**, 503-511 (2012).

4. Robertson, J.F.R., Di Leo, A., Fazal, M., Lichfield, J. & Ellis, M.J. Fulvestrant for hormone receptor-positive advanced breast cancer in patients with visceral versus non-visceral metastases: Findings from FALCON, FIRST, AND CONFIRM. *Cancer Res.* **78**, PD5–09 (2018).

5. Finn, R.S. et al. The cyclin-dependent kinase 4/6 inhibitor palbociclib in combination with letrozole versus letrozole alone as first-line treatment of oestrogen receptor-positive, HER2-negative, advanced breast cancer (PALOMA-1/TRIO-18): a randomised phase 2 study. *Lancet Oncol.* **16**, 25-35 (2015)

6. Finn, R.S. et al. Palbociclib and letrozole in advanced breast cancer. *N. Engl. J. Med.* **375**, 1925-1936 (2016).

7. Turner, N.C. et al. Overall survival with palbociclib and fulvestrant in advanced breast cancer. *N. Engl. J. Med.* **379**, 1926-1936 (2018).

8. Turner, N.C. et al. Palbociclib in hormone-receptor-positive advanced breast cancer. *N. Engl. J. Med.* **373**, 209-219 (2015).

9. Sledge, G.W. Jr. et al. The effect of abemaciclib plus fulvestrant on overall survival in hormone receptor-positive, ERBB2-negative breast cancer that progressed on endocrine therapy-MONARCH 2: a randomized clinical trial. *JAMA Oncol.* **6**, 116–124 (2020).

10. Slamon, D.J. et al. Overall survival (OS) results of the Phase III MONALEESA-3 trial of postmenopausal patients (pts) with hormone receptor-positive (HR+), human epidermal growth factor 2-negative (HER2−) advanced breast cancer (ABC) treated with fulvestrant (FUL) ± ribociclib (RIB). *Ann. Oncol.* **30**, v851-v934 (2019).

11. Slamon, D.J. et al. Phase III randomized study of ribociclib and fulvestrant in hormone receptor-positive, human epidermal growth factor receptor 2-negative advanced breast cancer: MONALEESA-3. *J. Clin. Oncol.* **36**, 2465-2472 (2018).
