# Supplementary material for: Meta-analyses of visceral versus non-visceral metastatic hormone receptor-positive breast cancer treated by endocrine monotherapies
Source: NPJ Breast Cancer. 2021 Feb 12;7:11. doi: 10.1038/s41523-021-00222-y (PMC7881093; doi:10.1038/s41523-021-00222-y)
Supplement: Supplementary file 2 — Reporting Summary Checklist [file 41523_2021_222_MOESM2_ESM.pdf]

nature research

Corresponding author(s): JOHN ROBERTSON NPJBCANCER-00740R

Last updated by author(s): Sep 15, 2020

Reporting Summary

Nature Research wishes to improve the reproducibility of the work that we publish. This form provides structure for consistency and transparency in reporting. For further information on Nature Research policies, see our Editorial Policies and the Editorial Policy Checklist.

Statistics

For all statistical analyses, confirm that the following items are present in the figure legend, table legend, main text, or Methods section.

n/a	Confirmed	
☐	☒	The exact sample size (*n*) for each experimental group/condition, given as a discrete number and unit of measurement
☒	☐	A statement on whether measurements were taken from distinct samples or whether the same sample was measured repeatedly
☐	☒	The statistical test(s) used AND whether they are one- or two-sided<br>*Only common tests should be described solely by name; describe more complex techniques in the Methods section.*
☐	☒	A description of all covariates tested
☐	☒	A description of any assumptions or corrections, such as tests of normality and adjustment for multiple comparisons
☐	☒	A full description of the statistical parameters including central tendency (e.g. means) or other basic estimates (e.g. regression coefficient) AND variation (e.g. standard deviation) or associated estimates of uncertainty (e.g. confidence intervals)
☐	☒	For null hypothesis testing, the test statistic (e.g. *F*, *t*, *r*) with confidence intervals, effect sizes, degrees of freedom and *P* value noted<br>*Give P values as exact values whenever suitable.*
☒	☐	For Bayesian analysis, information on the choice of priors and Markov chain Monte Carlo settings
☒	☐	For hierarchical and complex designs, identification of the appropriate level for tests and full reporting of outcomes
☐	☒	Estimates of effect sizes (e.g. Cohen's *d*, Pearson's *r*), indicating how they were calculated

Our web collection on statistics for biologists contains articles on many of the points above.

Software and code

Policy information about availability of computer code

Data collection	This manuscript presents the results of meta analyses. The individual patient data and aggregate data shared with the University of Nottingham (UON) were specific to the data requested for analysis and in accordance to the agreement and terms agreed between UON and providers of the data.
Data analysis	Data analysis was performed using standard R (v3.5.3) functions from the 'Metafor' package and standard SAS (v9.4) survival functions in particular PROC PHREG and PROC GLIMMIX.

For manuscripts utilizing custom algorithms or software that are central to the research but not yet described in published literature, software must be made available to editors and reviewers. We strongly encourage code deposition in a community repository (e.g. GitHub). See the Nature Research guidelines for submitting code & software for further information.

Data

Policy information about availability of data

All manuscripts must include a data availability statement. This statement should provide the following information, where applicable:

- Accession codes, unique identifiers, or web links for publicly available datasets
- A list of figures that have associated raw data
- A description of any restrictions on data availability

Data underlying the findings described in this manuscript may be obtained upon request as long as the request is in keeping with the terms under which the University of Nottingham received the data

Field-specific reporting

Please select the one below that is the best fit for your research. If you are not sure, read the appropriate sections before making your selection.

☒ Life sciences ☐ Behavioural & social sciences ☐ Ecological, evolutionary & environmental sciences

For a reference copy of the document with all sections, see nature.com/documents/nr-reporting-summary-flat.pdf

Life sciences study design

All studies must disclose on these points even when the disclosure is negative.

Sample size	This is a meta-analyses of phase 3 randomised clinical trials. The sample size is therefore dependent on the size of the original trials and also the % of patients within the trials who had hormone receptor positive tumors.
Data exclusions	Patients whose tumors were hormone receptor (HR) unknown or HR negative were excluded. The rationale for this is that if you are looking to judge the efficacy of endocrine therapy this is best assessed in the population of tumors which express the target for that therapy. Prior research has shown reduced or no effect when tumors are HR unknown or negative respectively. This was a pre-specified exclusion in this meta-analysis.
Replication	N/A
Randomization	The data was extracted from a number of randomised clinical trials. After excluding the HR unknown and negative tumors the analysis looked at the following pre-specified patient groups - 1) non-visceral metastases (non-VM), 2) visceral metastases (VM). The visceral metastases was also sub-divided into visceral liver metastases (VLM) and visceral non-liver metastases (VnLM).
Blinding	This is a meta-analysis and so the authors were not carrying out a clinical trial All the exclusions, sub-groups and data analyses were pre-specified and so blinding was not deemed appropriate.

Reporting for specific materials, systems and methods

We require information from authors about some types of materials, experimental systems and methods used in many studies. Here, indicate whether each material, system or method listed is relevant to your study. If you are not sure if a list item applies to your research, read the appropriate section before selecting a response.

Materials & experimental systems

n/a	Involved in the study	
☒	☐	Antibodies
☒	☐	Eukaryotic cell lines
☒	☐	Palaeontology and archaeology
☒	☐	Animals and other organisms
☒	☐	Human research participants
☐	☒	Clinical data
☒	☐	Dual use research of concern

Methods

n/a	Involved in the study	
☒	☐	ChIP-seq
☒	☐	Flow cytometry
☒	☐	MRI-based neuroimaging

Clinical data

Policy information about clinical studies

All manuscripts should comply with the ICMJE guidelines for publication of clinical research and a completed CONSORT checklist must be included with all submissions.

Clinical trial registration	This was not a clinical trial but a meta-analysis of data from a number of trials
Study protocol	N/A - this was not a clinical trial and therefore there was no clinical study protocol. The meta-analysis methodology is described in the manuscript.
Data collection	Data was obtained through a collaboration between researchers who had carried out and previously reported their individual studies and who had agreed to contribute data to address the specific question of the meta-analysis.
Outcomes	The outcome measures were defined and pre-specified - as described in the manuscript.
